# Supplementary material for: The natural pyrazolotriazine pseudoiodinine from Pseudomonas mosselii 923 inhibits plant bacterial and fungal pathogens
Source: Nat Commun. 2023 Feb 9;14:734. doi: 10.1038/s41467-023-36433-z (PMC9911603; doi:10.1038/s41467-023-36433-z)
Supplement: Supplementary file 1 — Supplementary Information [file 41467_2023_36433_MOESM1_ESM.pdf]

Supplementary Information

The natural pyrazolotriazine pseudoiodinine from *Pseudomonas mosselii* 923 inhibits plant bacterial and fungal pathogens

Ruihuan Yang[1,#], Qing Shi[2,#], Tingting Huang[2], Yichao Yan[1], Shengzhang Li[1], Yuan Fang[1], Ying Li[1], Linlin Liu[1], Longyu Liu[1], Xiaozheng Wang[2], Yongzheng Peng[1], Jiangbo Fan[1], Lifang Zou[1,2]*, Shuangjun Lin[2], and Gongyou Chen[1,2]*

[1] Shanghai Collaborative Innovation Center of Agri-Seeds/School of Agriculture and Biology, Shanghai Jiao Tong University, Shanghai 200240, China

[2] State Key Laboratory of Microbial Metabolism, School of Life Sciences & Biotechnology, Shanghai Jiao Tong University, Shanghai 200240, China

[#] These authors contributed equally: Ruihuan Yang, Qing Shi.

*Corresponding authors: zoulifang202018@sjtu.edu.cn; gyouchen@sjtu.edu.cn

Supplementary Figures

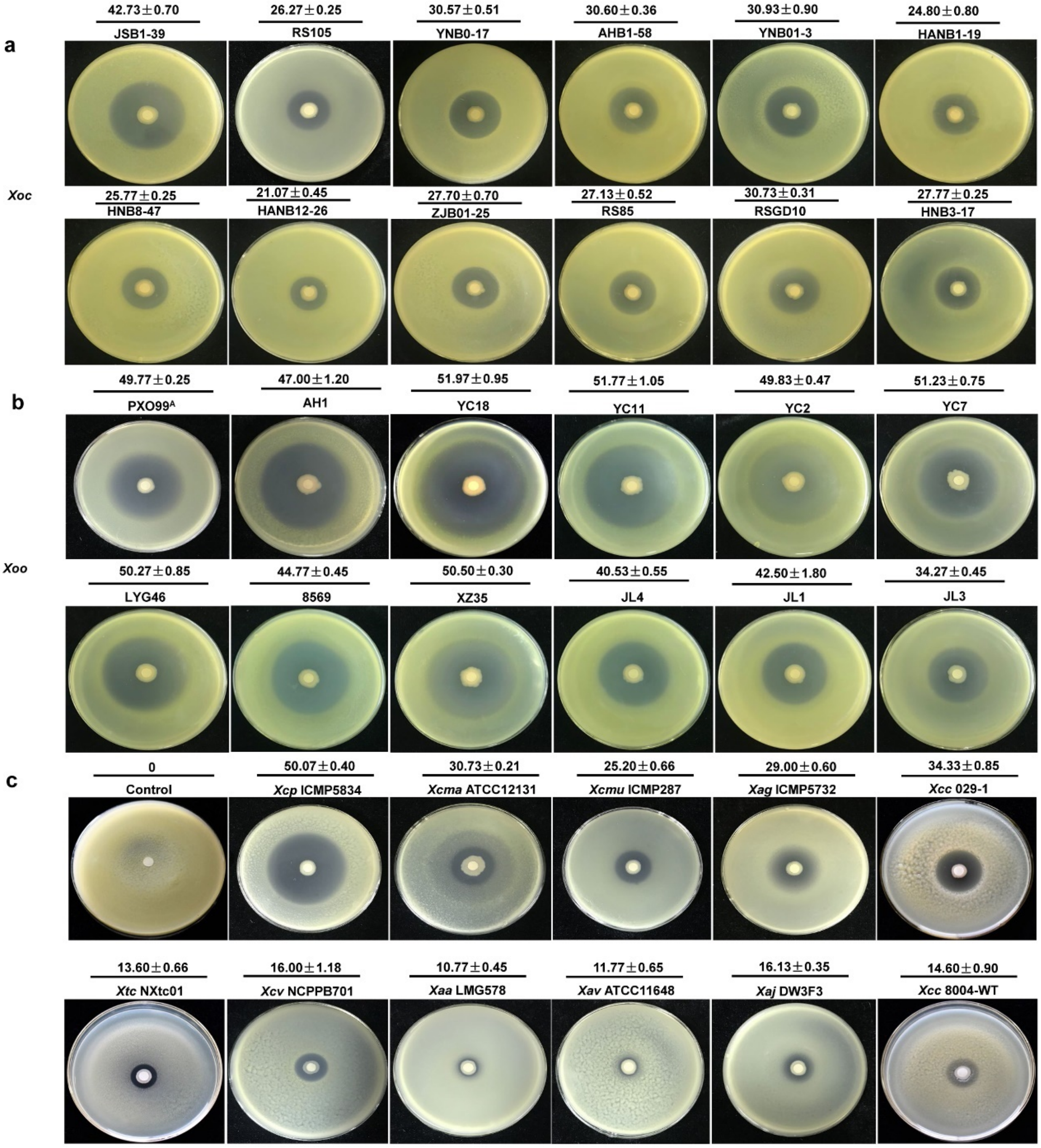

Supplementary Figure 1. Antimicrobial activity of *P. mosselii* 923 when co-cultivated with various *Xanthomonas* strains. a*, Xoc* strains included: RS105, JSB1-39, AHB1-58, YNB01-3, YNB0-17, HNB8-47, ZJB01-25, HNB3-17, RS85, HANB1-19, HANB12-26 and RSGD10. **b***, Xoo* strains included: PXO99^{A}, AH1, YC18, XZ35, LYG46, YC2, YC7, YC11, 8569, JL1, JL4 and JL3. **c***,* Other *Xanthomonas* spp. included *X. campestris* pv. *phaseoli, Xcp* ICMP5834; *X. campestris* pv. *malvacearum, Xcma* ATCC12131: *X. campestris* pv. *musacearum, Xcmu* ICMP287; *X. axonopodis* pv. *glycines, Xag* ICMP5732; *X. citri subsp. citri, Xcc* 029-1; *X. translucens* pv. *cereali, Xtc* NXtc01; *X. campestris* pv. *vesicatoria, Xcv* NCPPB701; *X. axonopodis* pv. *allii, Xaa* LMG578; *X. axonopodis* pv. *vignicola, Xav* ATCC11648; *X. arboricola* pv. *juglandis, Xaj* DW3F3; and *X. campestris* pv. *campestris, Xcc* 8004-WT. The untreated plate was used for control. Values represent the mean diameter of inhibition zones ± SD (mm) (*n*=3 independent plates). Three independent biological experiments were performed with similar results.

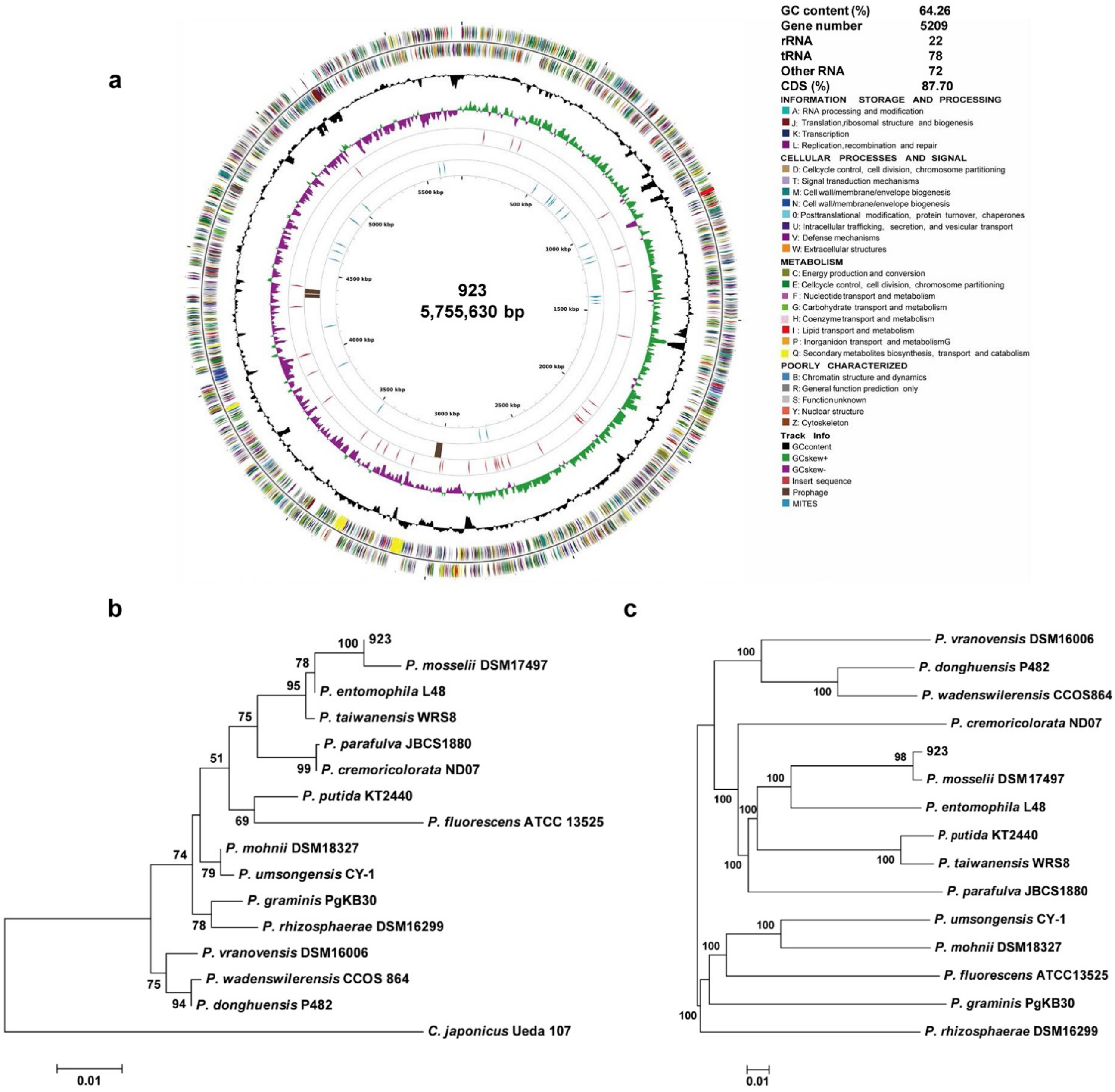
a
GC content (%) 64.26
Gene number 5209
rRNA 22
tRNA 78
Other RNA 72
CDS (%) 87.70
INFORMATION STORAGE AND PROCESSING
A: RNA processing and modification
J: Translation,ribosomal structure and biogenesis
K: Transcription
L: Replication,recombination and repair
CELLULAR PROCESSES AND SIGNAL
D: Cellcycle control, cell division, chromosome partitioning
T: Signal transduction mechanisms
M: Cell wall/membrane/envelope biogenesis
N: Cell wall/membrane/envelope biogenesis
0: Posttranslational modification, protein turnover, chaperones
U: Intracellular trafficking, secretion, and vesicular transport
V: Defense mechanisms
W: Extracellular structures
METABOLISM
C: Energy production and conversion
E: Cellcycle control, cell division, chromosome partitioning
F: Nucleotide transport and metabolism
G: Carbohydrate transport and metabolism
H: Coenzyme transport and metabolism
I : Lipid transport and metabolism
P : Inorganicion transport and metabolismG
Q: Secondary metabolites biosynthesis, transport and catabolism
POORLY CHARACTERIZED
B: Chromatin structure and dynamics
R: General function prediction only
S: Functionunknown
Y: Nuclear structure
Z: Cytoskeleton
Track Info
GCcontent
GCskew+
GCskew-
Insert sequence
Prophage
MITES
923
5,755,630 bp
b
923
P. mosselii DSM17497
P. entomophila L48
P. taiwanensis WRS8
P. parafulva JBCS1880
P. cremoricolorata ND07
P. putida KT2440
P. fluorescens ATCC 13525
P. mohnii DSM18327
P. umsongensis CY-1
P. graminis PgKB30
P. rhizosphaerae DSM16299
P. vranovensis DSM16006
P. wadenswilerensis CCOS 864
P. donghuensis P482
C. japonicus Ueda 107
0.01
c
P. vranovensis DSM16006
P. donghuensis P482
P. wadenswilerensis CCOS864
P. cremoricolorata ND07
923
P. mosselii DSM17497
P. entomophila L48
P. putida KT2440
P. taiwanensis WRS8
P. parafulva JBCS1880
P. umsongensis CY-1
P. mohnii DSM18327
P. fluorescens ATCC13525
P. graminis PgKB30
P. rhizosphaerae DSM16299
0.01

Supplementary Figure 2. Identification of the pseudoiodinine-producing strain 923 as *P. mosselii*. **a**, Circular genomic map of strain 923. The outermost and second outermost circles indicate CDS (coding sequences) on forward and reverse strands and are color-coded according to COG (clusters of orthologous groups) categories. The fourth circle shows G + C content and the G + C skew in green (+) and purple (−), respectively. The fifth circle shows insertion sequences in red, putative prophage remnants in brown, and miniature inverted repeats / transposable elements (MITEs) in blue. The scale is shown in the innermost circle. The GC content of the chromosome was 64.26% and contained 5,441 (87.7%) protein coding sequences (CDSs), 22 rRNA genes, 78 tRNA genes, and 72 other noncoding RNA genes. Strain 923 lacked plasmid DNA. **b**, Neighbor-joining phylogenetic tree based on the sequence of *16S rRNA* from strain 923 and closely-related *Pseudomonas* spp. Bootstrap values > 50% of 1000 replicates are shown at the nodes. *Cellvibrio japonicus* Ueda 107 was used as an outgroup. **c**, Phylogenetic tree based on the genomic sequence of 923 and 14 fully-sequenced *Pseudomonas* strains.

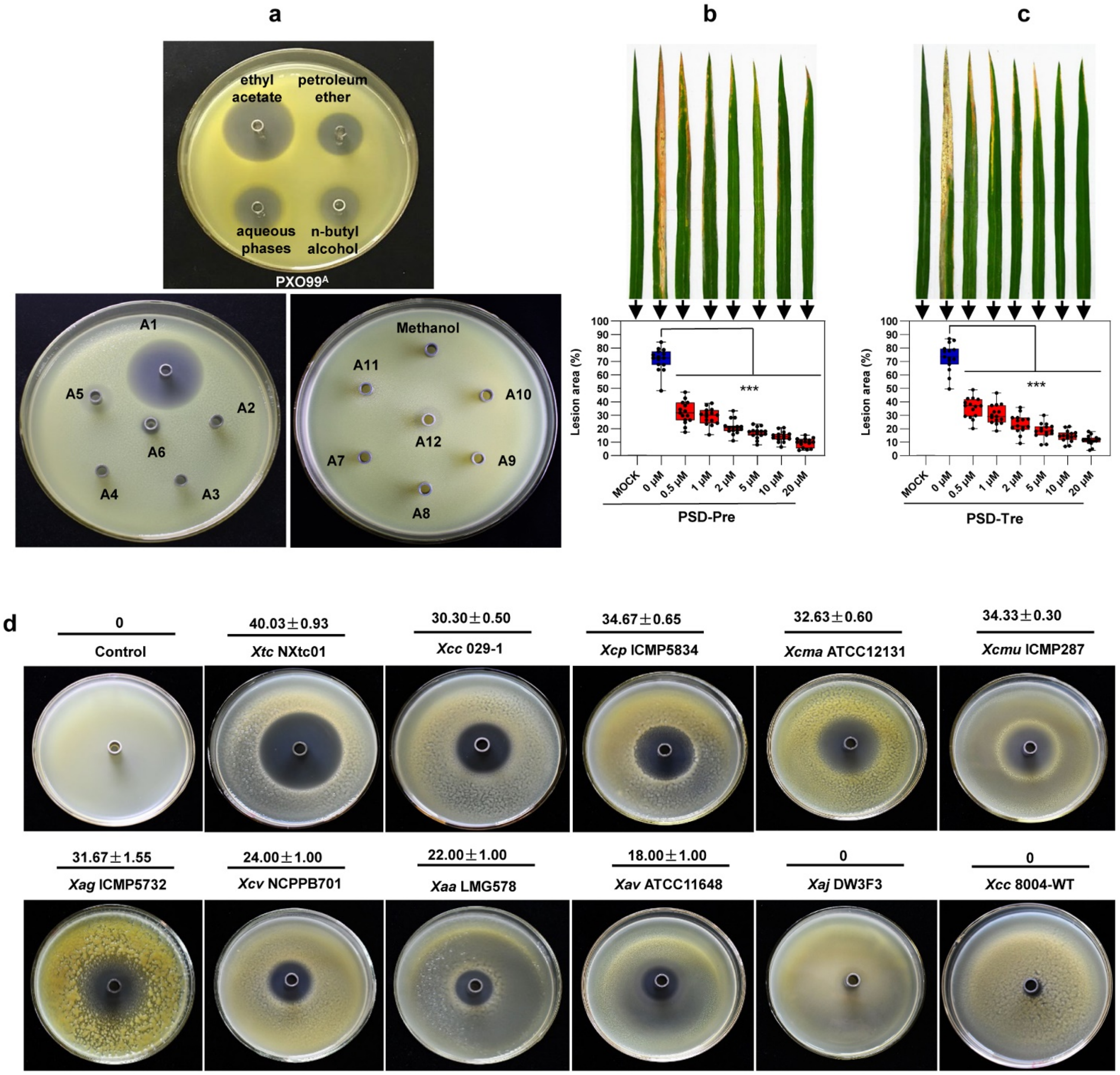
a
ethyl acetate
petroleum ether
aqueous phases
n-butyl alcohol
PXO99A
A1
A2
A3
A4
A5
A6
Methanol
A7
A8
A9
A10
A11
A12
b
c
Lesion area (%)
***
MOCK
0 μM
0.5 μM
1 μM
2 μM
5 μM
10 μM
20 μM
PSD-Pre
PSD-Tre
d
0
Control
40.03±0.93
Xtc NXtc01
30.30±0.50
Xcc 029-1
34.67±0.65
Xcp ICMP5834
32.63±0.60
Xcma ATCC12131
34.33±0.30
Xcmu ICMP287
31.67±1.55
Xag ICMP5732
24.00±1.00
Xcv NCPPB701
22.00±1.00
Xaa LMG578
18.00±1.00
Xav ATCC11648
0
Xaj DW3F3
0
Xcc 8004-WT

Supplementary Figure 3. Isolation of metabolite responsible for antimicrobial activity in strain 923. a, Antimicrobial activity of the aqueous phase and extracts from petroleum ether, ethyl acetate, *n*-butyl alcohol and fractions A1-A12. Antimicrobial activity is shown on agar seeded with *Xoo* PXO99^A; methanol was used as the control. **b**, **c**, Efficacy of pseudoiodinine (PSD) for control of *Xoo* PXO99^A in the field. Pseudoiodinine was applied at concentrations of 0, 0.5, 1, 2, 5, 10 and 20 μM, and lesion areas were measured at 15 dpi. Panel **b** shows symptoms on rice leaves sprayed with pseudoiodinine 12 h prior to inoculation with *Xoo* PXO99^A (PSD-Pre); panel **c** shows symptoms on rice 12 h after inoculation with *Xoo* PXO99^A followed by pseudoiodinine treatment (PSD-Tre). Data points represent means ± SD (n=15 independent leaves). The significant differences at ***$P < 0.001$, ***$P = 8.3\times10^{-33}$, $= 3.4\times10^{-35}$, $= 3.3\times10^{-41}$, $= 4.1\times10^{-44}$, $= 6.0\times10^{-46}$, $= 7.0\times10^{-49}$ in sequence of **b**; ***$P < 0.001$, ***$P = 2.8\times10^{-27}$, $= 3.7\times10^{-31}$, $= 3.5\times10^{-36}$, $= 1.4\times10^{-39}$, $= 4.7\times10^{-42}$, $= 6.5\times10^{-44}$ in sequence of **c.** The centre bar represents the mean, and Min to Max of box and whiskers was used. Statistical significance was determined using the LSD test method with one-way ANOVA. The experiment was repeated three times independently with similar results. **d**, Analysis of pseudoiodinine activity for 11 *Xanthomonas* spp. Numbers above pictures show the mean diameters of inhibition zones ± SD (mm) (n=3 independent plates). Methanol was used for the control. Three independent biological experiments were performed with similar results.

Physical and Spectroscopic Data of pseudoiodinine. ^{1}H NMR (600 MHz, CD_3OD) δ 9.14 (s, 1H), 4.44 (s, 3H), 4.27 (s, 3H). ^{13}C NMR (150 MHz, CD_3OD) δ 161.0, 146.1, 144.3, 140.4, 57.7, 43.3. HRMS-ESI (*m/z*): $[M+H]^+$ calcd for $C_6H_8N_5O$, 166.0729; found, 166.0737.

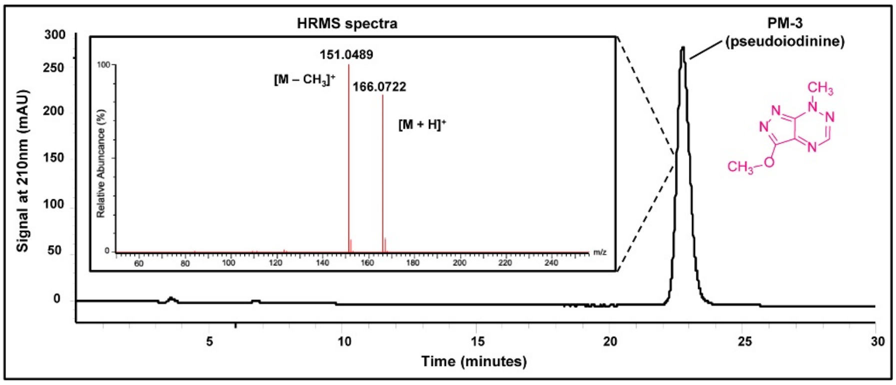

Supplementary Figure 4. Structural determination of PM-3 (pseudoiodinine). HPLC chromatogram (Method B: detected at 210 nm) of PM-3. Inset, high-resolution mass spectra (HRMS) of PM-3 showing a peak at m/z 166.0722, which corresponds to the $[M + H]^+$ ion and another at m/z 151.0489, which corresponds to $[M – CH_3]^+$ ion.

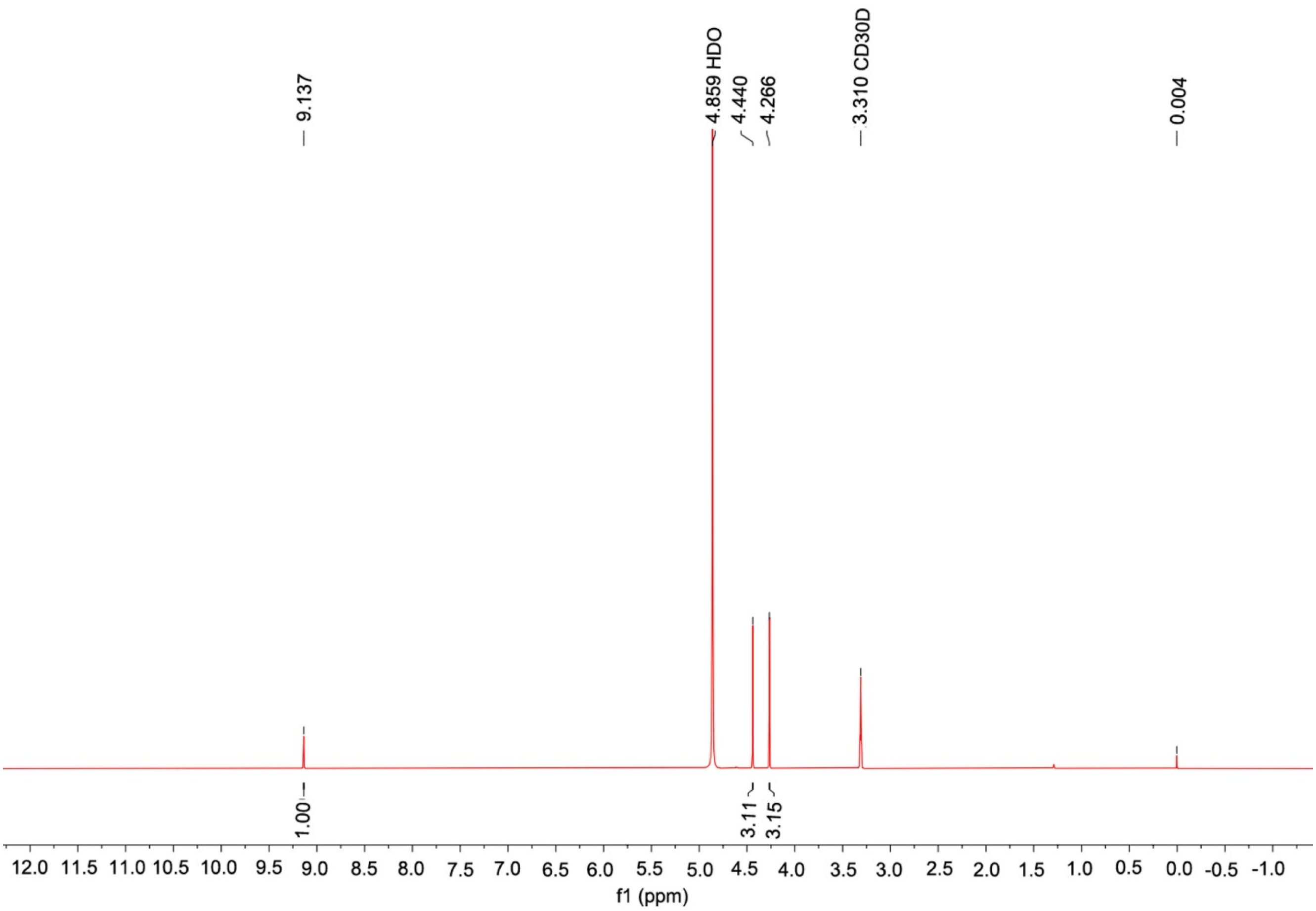

Supplementary Figure 5. Structural determination of PM-3 (pseudoiodinine). ^{1}H NMR spectrum of pseudoiodinine. ^{1}H NMR (600 MHz, CD_3OD) δ 9.14 (s, 1H), 4.44 (s, 3H), 4.27 (s, 3H).

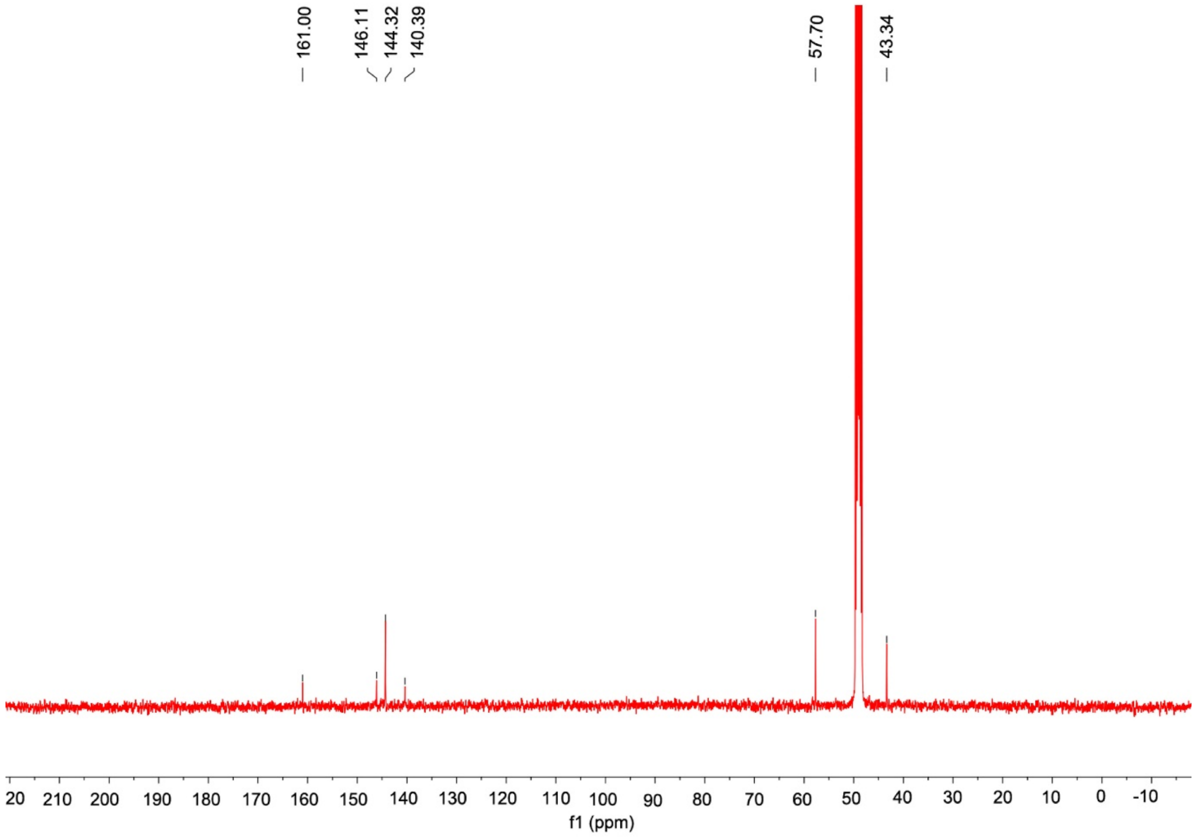

Supplementary Figure 6. Structural determination of PM-3 (pseudoiodinine). ^{13}C NMR spectrum of pseudoiodinine. ^{13}C NMR (150 MHz, CD_3OD) δ 161.0, 146.1, 144.3, 140.4, 57.7, 43.3.

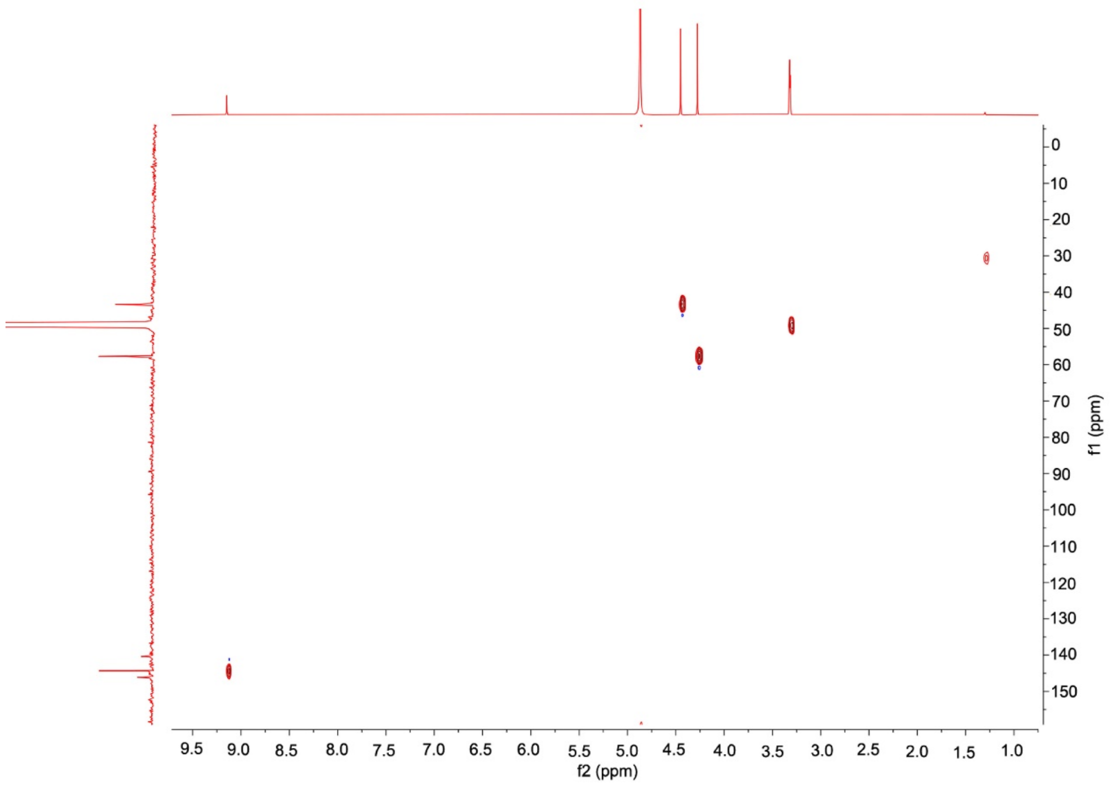

Supplementary Figure 7. Structural determination of PM-3 (pseudoiodinine). ^{1}H-^{13}C heteronuclear single quantum coherence (HSQC) NMR spectrum of pseudoiodinine. (^{1}H, 600 MHz, CD_3OD; ^{13}C, 150 MHz, CD_3OD).

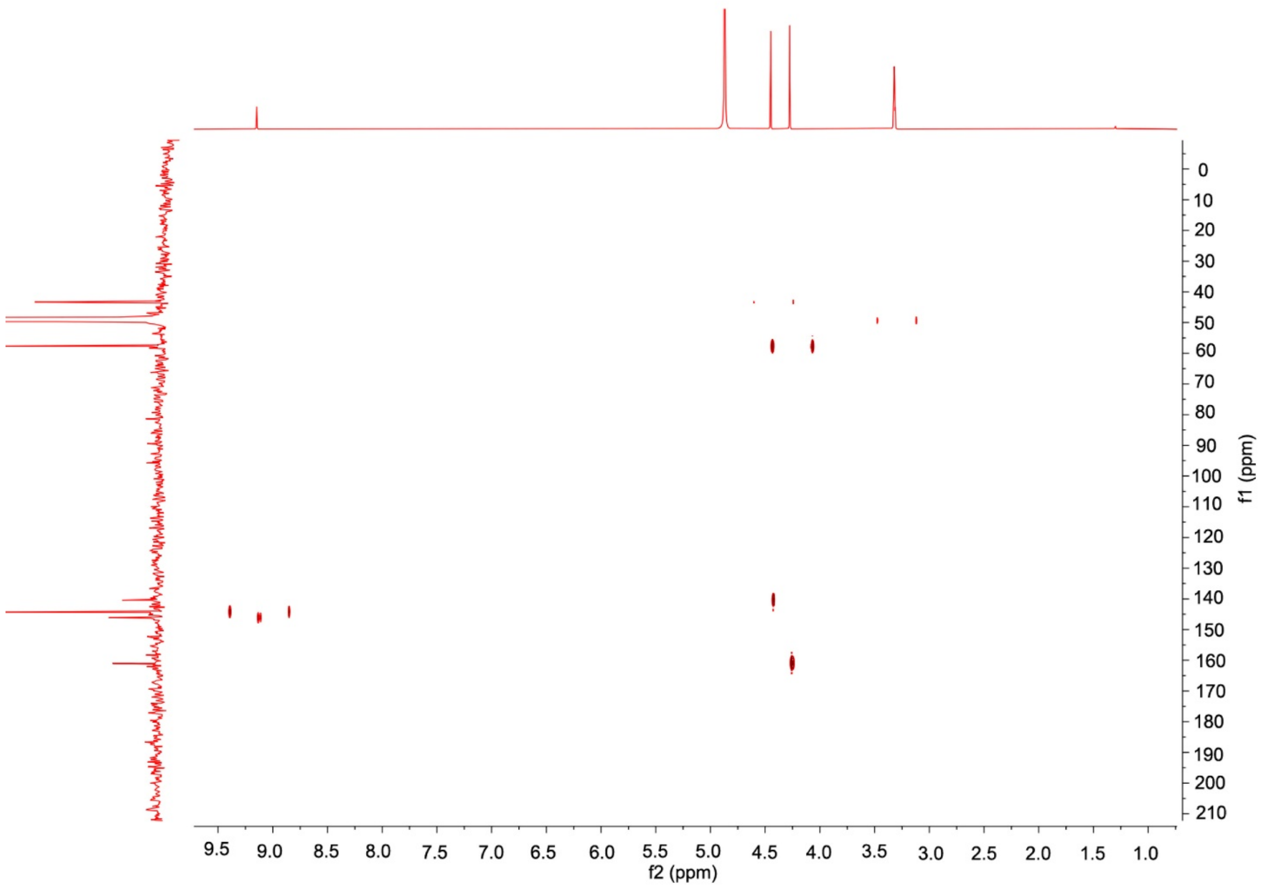

Supplementary Figure 8. Structural determination of PM-3 (pseudoiodinine). ^{1}H-^{13}C heteronuclear multiple bond correlation (HMBC) NMR spectrum of pseudoiodinine. (^{1}H, 600 MHz, CD_3OD; ^{13}C, 150 MHz, CD_3OD).

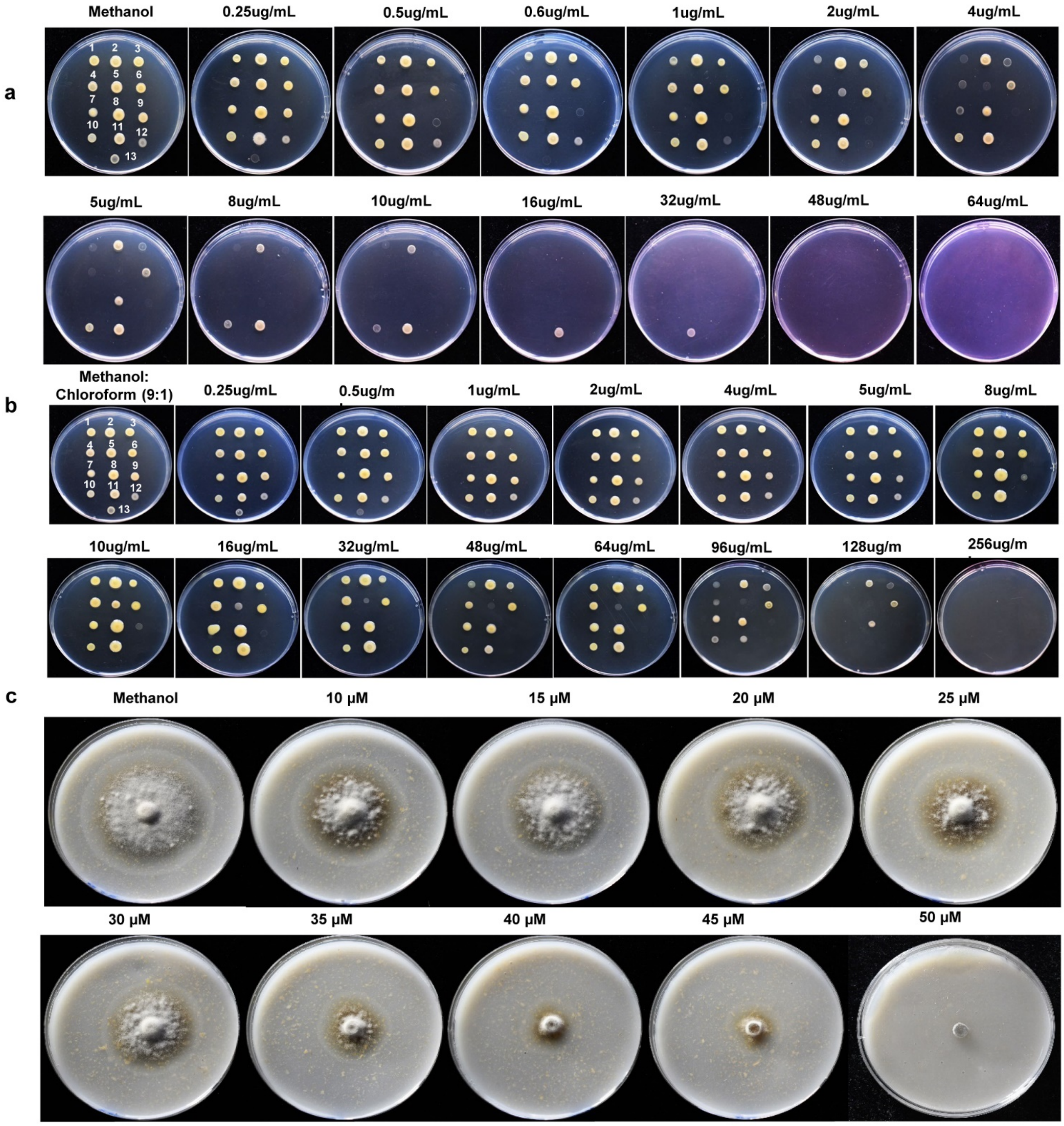
a
Methanol
0.25ug/mL
0.5ug/mL
0.6ug/mL
1ug/mL
2ug/mL
4ug/mL
1 2 3 4 5 6 7 8 9 10 11 12 13
5ug/mL
8ug/mL
10ug/mL
16ug/mL
32ug/mL
48ug/mL
64ug/mL
b
Methanol: Chloroform (9:1)
0.25ug/mL
0.5ug/m
1ug/mL
2ug/mL
4ug/mL
5ug/mL
8ug/mL
1 2 3 4 5 6 7 8 9 10 11 12 13
10ug/mL
16ug/mL
32ug/mL
48ug/mL
64ug/mL
96ug/mL
128ug/m
256ug/m
c
Methanol
10 μM
15 μM
20 μM
25 μM
30 μM
35 μM
40 μM
45 μM
50 μM

Supplementary Figure 9. Determination of minimum inhibitory concentrations (MICs) of pseudoiodinine and phenazine-1-carboxylic acid (PCA). a, Dilutions of 13 *Xanthomonas* strains were spotted onto NA containing methanol (control), and 0.25, 0.5, 0.6, 1, 2, 4, 5, 8, 10, 16, 32, 48 and 64 µg/mL pseudoiodinine. Strains tested include the following (left to right): spot no. 1, *X. campestris* pv. *musacearum* ICMP287; 2, *X. campestris* pv. *malvacearum* ATCC12131; 3, *X. axonopodis* pv. *glycines* ICMP5732; 4, *X. campestris* pv. *vesicatoria* NCPPB701; 5, *X. campestris* pv. *phaseoli* ICMP5834; 6, *X. axonopodis* pv. *vignicola* ATCC11648; 7, *X. citri subsp. citri* 029-1; 8, *X. axonopodis* pv. *allii* LMG578; 9, *X. translucens* pv. *cerealis* NXtc01; 10, *X. arboricola* pv. *juglandis* DW3F3; 11, *X. campestris pv. campestris* 8004-WT; 12, *Xoc* RS105; and 13, *Xoo* PXO99^{A}. **b,** Inhibition of 13 *Xanthomonas* strains [as named and numbered in panel (**a**)] on NA containing methanol:chloroform (9:1) (control) and PCA at 0.25, 0.5, 1, 2, 4, 5, 8, 10, 16, 32, 48, 64, 96, 128 and 256 µg/mL. **c**, Growth of *M. oryzae* R01-1 on OMA containing methanol (control) and 10, 15, 20, 25, 30, 35, 40, 45 and 50 µM pseudoiodinine. Three independent biological experiments were performed with similar results.

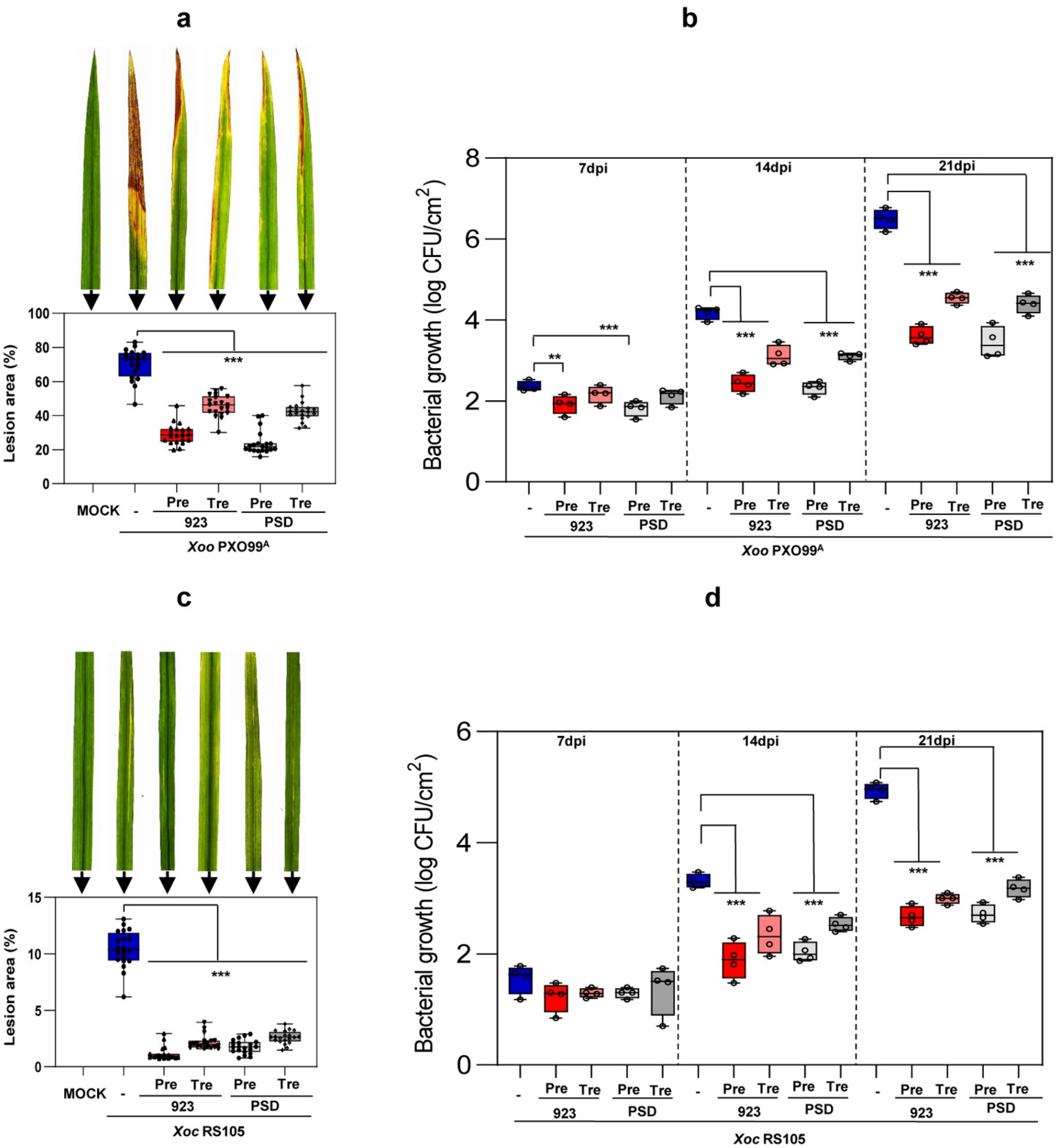
a
b
c
d
Lesion area (%)
MOCK
Pre
Tre
923
PSD
Xoo PXO99^A
Bacterial growth (log CFU/cm2)
7dpi
14dpi
21dpi
Xoc RS105

Supplementary Figure 10. Effects of *P. mosselii* 923 and pseudoiodinine on the growth inhibition of *Xoo* and *Xoc in planta*. **a,** Rice leaves sprayed with strain 923 (OD_{600}=0.5) or 0.5 μg/mL pseudoiodinine (PSD) 12 h before (Pre) and after (Tre) inoculation with *Xoo* PXO99^{A} (OD_{600}=0.6) in the greenhouse. Lesion areas were quantified from infected leaves at 15 d post-infection. Data points represent means ± SD (*n*=20 independent leaves). The significant differences at ****P* < 0.001, ****P*= 4.3×10^{-34}, = 4.1×10^{-19}, = 4.2×10^{-38}, = 2.0×10^{-22} in sequence. Lesion inoculation assays to determine the *Xoo* PXO99^{A} (**b**) and *Xoc* RS105 (**d**) population size at 7, 14 and 21 dpi. Data are shown as means ± SD (*n*=4 biologically independent samples). **b,** The significant differences at ****P* < 0.001, ***P* < 0.01, ***P*= 0.005, ****P*= 0.001 of 7dpi, ****P*= 1.1×10^{-9}, = 7.5×10^{-7}, = 4.5×10^{-10}, = 6.8×10^{-7} in sequence of 14 dpi, ****P*= 8.5×10^{-11}, = 1.9×10^{-8}, = 3.9×10^{-11}, = 7.2×10^{-9} in sequence of 21 dpi. **d,** The significant differences at ****P* < 0.001, ****P*= 6.1×10^{-7}, = 0.00005, = 0.000002, = 0.0004 in sequence of 14 dpi, ****P*= 1.4×10^{-12}, = 1.3×10^{-11}, = 1.9×10^{-12}, = 5.5×10^{-11} in sequence of 21 dpi. **c,** Rice leaves sprayed with strain 923 (OD_{600}=0.5) and 4 μg/mL PSD at 12 h before (Pre) and after (Tre) inoculation with *Xoc* RS105 (OD_{600}=0.6) in the greenhouse. Data points represent means ± SD (*n*=20 independent leaves). The significant differences at ****P* < 0.001, ****P*= 3.7×10^{-53}, = 6.9×10^{-49}, = 1.5×10^{-50}, = 7.7×10^{-47} in sequence. The centre bar represents the mean, and Min to Max of box and whiskers was used. Statistical significance was assessed by the LSD test method with one-way ANOVA. The experiments were repeated three times independently with similar results.

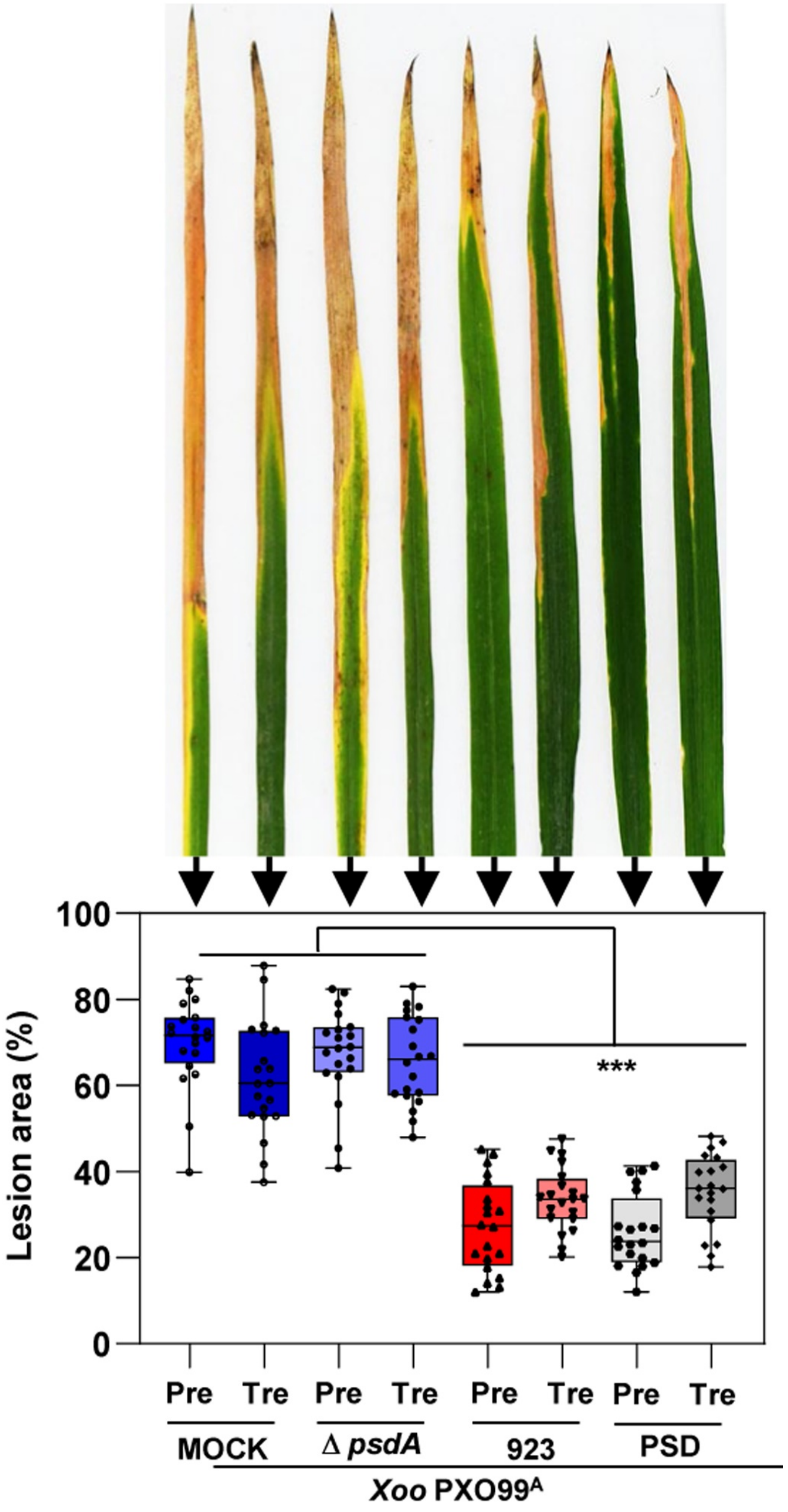
100
80
60
40
20
0
Lesion area (%)
***
Pre
Tre
Pre
Tre
Pre
Tre
Pre
Tre
MOCK
Δ psdA
923
PSD
Xoo PXO99A

Supplementary Figure 11. Effects of mutant Δ*psdA*, WT *P. mosselii* 923 and pseudoiodinine on growth inhibition of *Xoo in planta*. Rice leaves sprayed with strains Δ*psdA*/923 (OD_{600} = 0.5) or 0.5 μg/mL pseudoiodinine (PSD) 12 h before (Pre) and after (Tre) inoculation with *Xoo* PXO99^{A} (OD_{600} = 0.6) in the greenhouse. Lesion areas were quantified from infected leaves at 21 d post-infection. Data points represent means ± SD (n=20 independent leaves). The significant differences at ***$P < 0.001$, ***P= 3.1×10^{-32}, = 1.1×10^{-25}, = 1.3×10^{-33}, = 5.3×10^{-24} in sequence. The centre bar represents the mean, and Min to Max of box and whiskers was used. Statistical significance was assessed by the LSD test method with one-way ANOVA. Experiments were repeated three times independently with similar results.

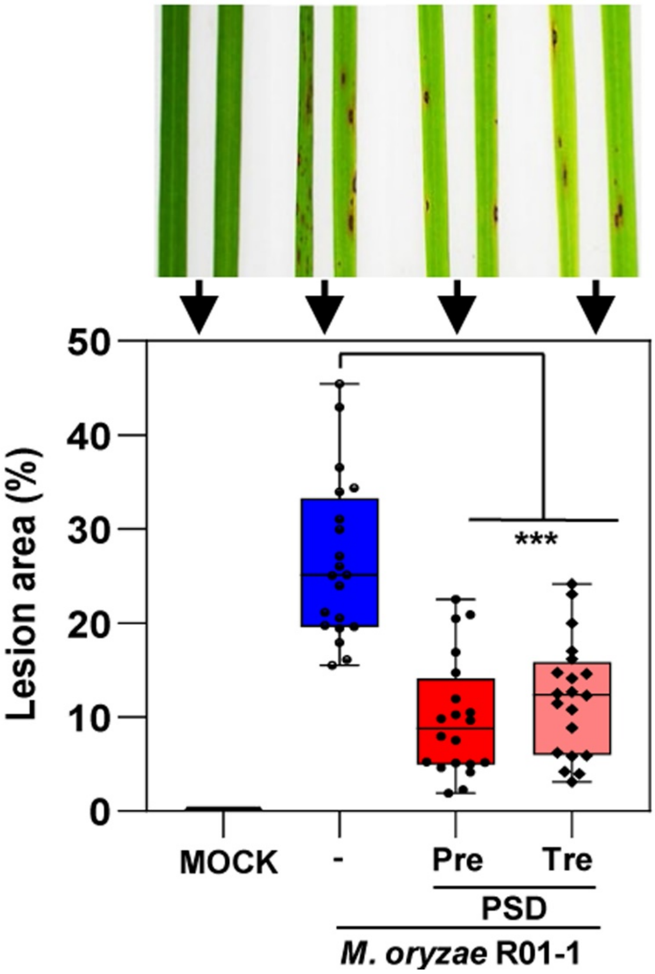

Supplementary Figure 12. Effects of pseudoiodinine on rice leaf infection by *M. oryzae*. Pseudoiodinine (PSD) was sprayed onto the surface of rice leaves at a concentration of 40 μM at 24 h before (Pre) and after (Tre) inoculation with *M. oryzae* R01-1 spore. The rice blast disease lesion area was calculated from infected rice leaves 7 d post-infection. Data points represent means ± SD (*n*=20 independent leaves). The significant differences at $***P < 0.001$, $***P= 5.5\times10^{-10}$, $= 2.6\times10^{-8}$ in sequence. The centre bar represents the mean, and Min to Max of box and whiskers was used. Statistical significance was assessed by the LSD test method with one-way ANOVA. Experiments were repeated three times independently with similar results.

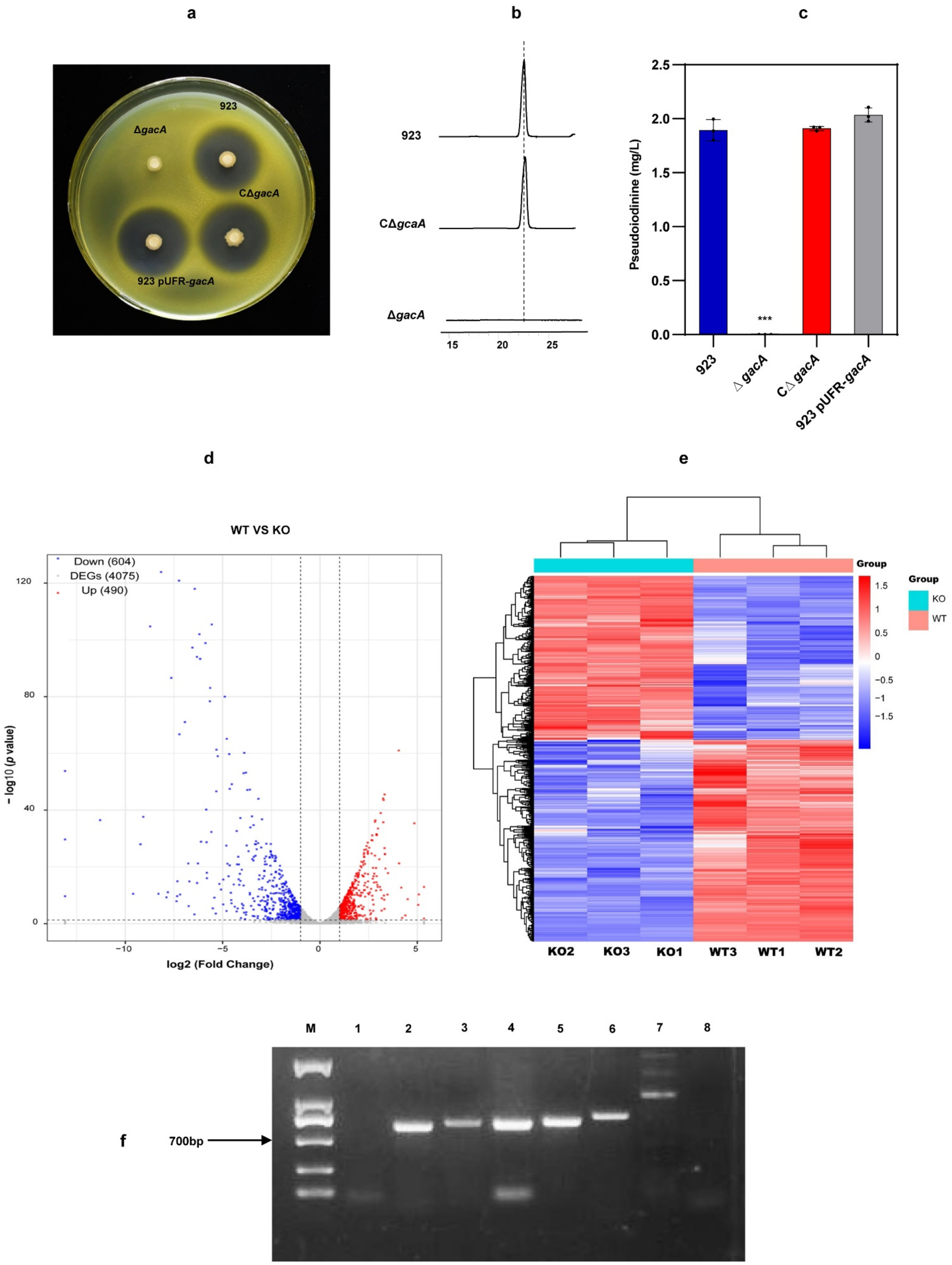

M: marker 2: *psdA-psdB* 3: *psdB-psdC* 4: *psdC-psdD* 5:*psdD-psdE* 6:*psdE-psdF*
7: *psdF-psdG* (1 and 8 are controls)

Supplementary Figure 13. Screening of pseudoiodinine synthetic genes. a, Antibacterial activity of *P. mosselii* 923, the Δ*gacA* mutant, complemented strain CΔ*gacA*, and strain 923 containing pUFR-*gacA* (overexpressing strain). **b,** HPLC profiles (Method B, detected at 500 nm) of 923, the Δ*gacA* mutant and complemented strain 923 pUFR-*gacA*. **c,** Pseudoiodinine production in *P. mosselii* 923, the Δ*gacA* mutant, complemented strain CΔ*gacA*, and overexpressing strain 923 pUFR-*gacA*. Error bars show means ± SD (n = 3 independent experiments) and significant differences at ***, $P < 0.001$, ***$P = 1.1\times10^{-8}$ (one-way ANOVA followed by LSD test). **d**, **e**, Transcriptome analysis of WT (923) and KO (Δ*gacA*) strains of *P. mosselii*. Panel **d,** Volcano plots showing differentially-expressed genes (DEGs). Red and blue dots represent significantly up- and down-regulated DEGs; grey dots are not significant. Results were deemed significant if |log2(FC)| ≥ 1 and FDR-corrected $P < 0.005$, two-tailed unpaired Student's t-test. **e,** Heat map showing differentially expressed genes (|log2(FC)|); red indicates upregulation and green indicates downregulation. **f**, Intergenic PCR analysis of the pseudoiodinine biosynthetic gene cluster, the cDNA of *P. mosselii* 923 was used as the template. Lanes: M, molecular weight marker; 2, *psdA-psdB*; 3, *psdB-psdC*; 4, *psdC-psdD*; 5, *psdD-psdE*; 6, *psdE-psdF*; 7, *psdF-psdG*.; lanes 1 and 8 are controls). Experiment were repeated three times independently with similar results.

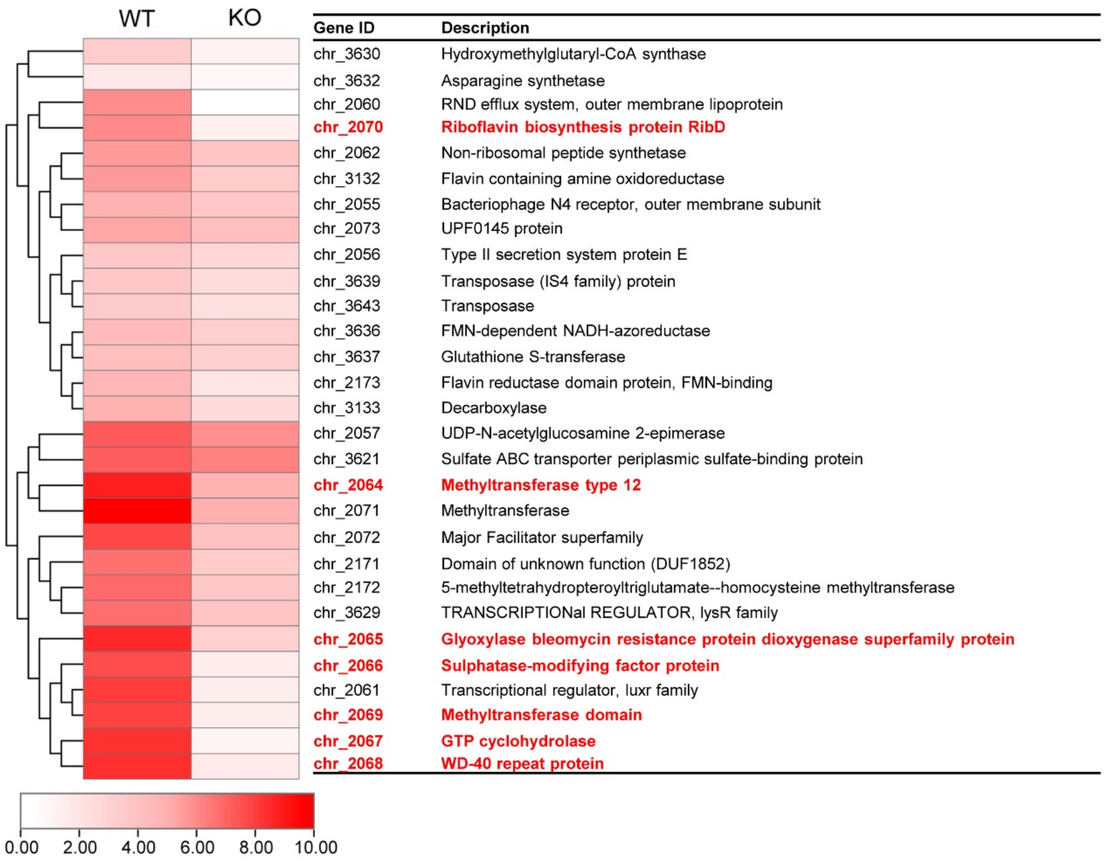

Gene ID	Description
chr_3630	Hydroxymethylglutaryl-CoA synthase
chr_3632	Asparagine synthetase
chr_2060	RND efflux system, outer membrane lipoprotein
**chr_2070**	**Riboflavin biosynthesis protein RibD**
chr_2062	Non-ribosomal peptide synthetase
chr_3132	Flavin containing amine oxidoreductase
chr_2055	Bacteriophage N4 receptor, outer membrane subunit
chr_2073	UPF0145 protein
chr_2056	Type II secretion system protein E
chr_3639	Transposase (IS4 family) protein
chr_3643	Transposase
chr_3636	FMN-dependent NADH-azoreductase
chr_3637	Glutathione S-transferase
chr_2173	Flavin reductase domain protein, FMN-binding
chr_3133	Decarboxylase
chr_2057	UDP-N-acetylglucosamine 2-epimerase
chr_3621	Sulfate ABC transporter periplasmic sulfate-binding protein
**chr_2064**	**Methyltransferase type 12**
chr_2071	Methyltransferase
chr_2072	Major Facilitator superfamily
chr_2171	Domain of unknown function (DUF1852)
chr_2172	5-methyltetrahydropteroyltriglutamate--homocysteine methyltransferase
chr_3629	TRANSCRIPTIONal REGULATOR, lysR family
**chr_2065**	**Glyoxylase bleomycin resistance protein dioxygenase superfamily protein**
**chr_2066**	**Sulphatase-modifying factor protein**
chr_2061	Transcriptional regulator, luxr family
**chr_2069**	**Methyltransferase domain**
**chr_2067**	**GTP cyclohydrolase**
**chr_2068**	**WD-40 repeat protein**

Supplementary Figure 14. Comparison of selected upregulated (|log2(FC)|) genes in WT (923) and KO (Δ*gacA*) strains. Genes that are highly upregulated in the WT are designated in red font.

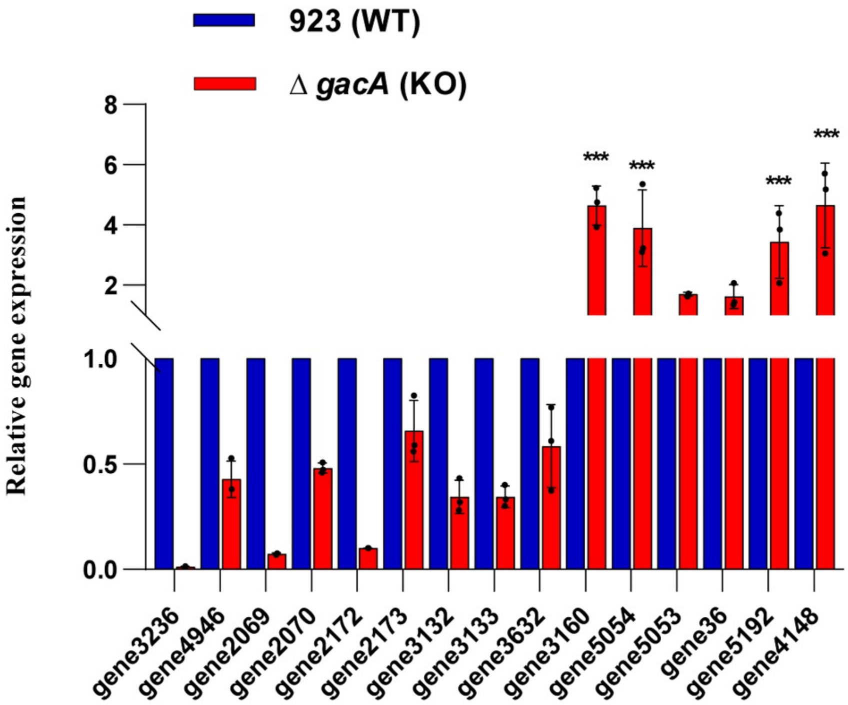

Supplementary Figure 15. Expression of *gene3236*, *gene4946*, *gene2069*, *gene2070*, *gene2172*, *gene2173*, *gene3132*, *gene3133*, *gene3632*, *gene3160*, *gene5054*, *gene5053*, *gene36*, *gene5192* and *gene4148* in Δ*gacA* (KO) compared to *P. mosselii* 923 (WT). Error bars show means ± SD (n = 3 biological independent replicates) and significant differences at $***P < 0.001$, $***P = 1.7\times10^{-8}$, = 0.000001, = 0.00002, = 1.5×10^{-8} in sequence (one-way ANOVA followed by LSD test). Experiments were repeated three times independently with similar results.

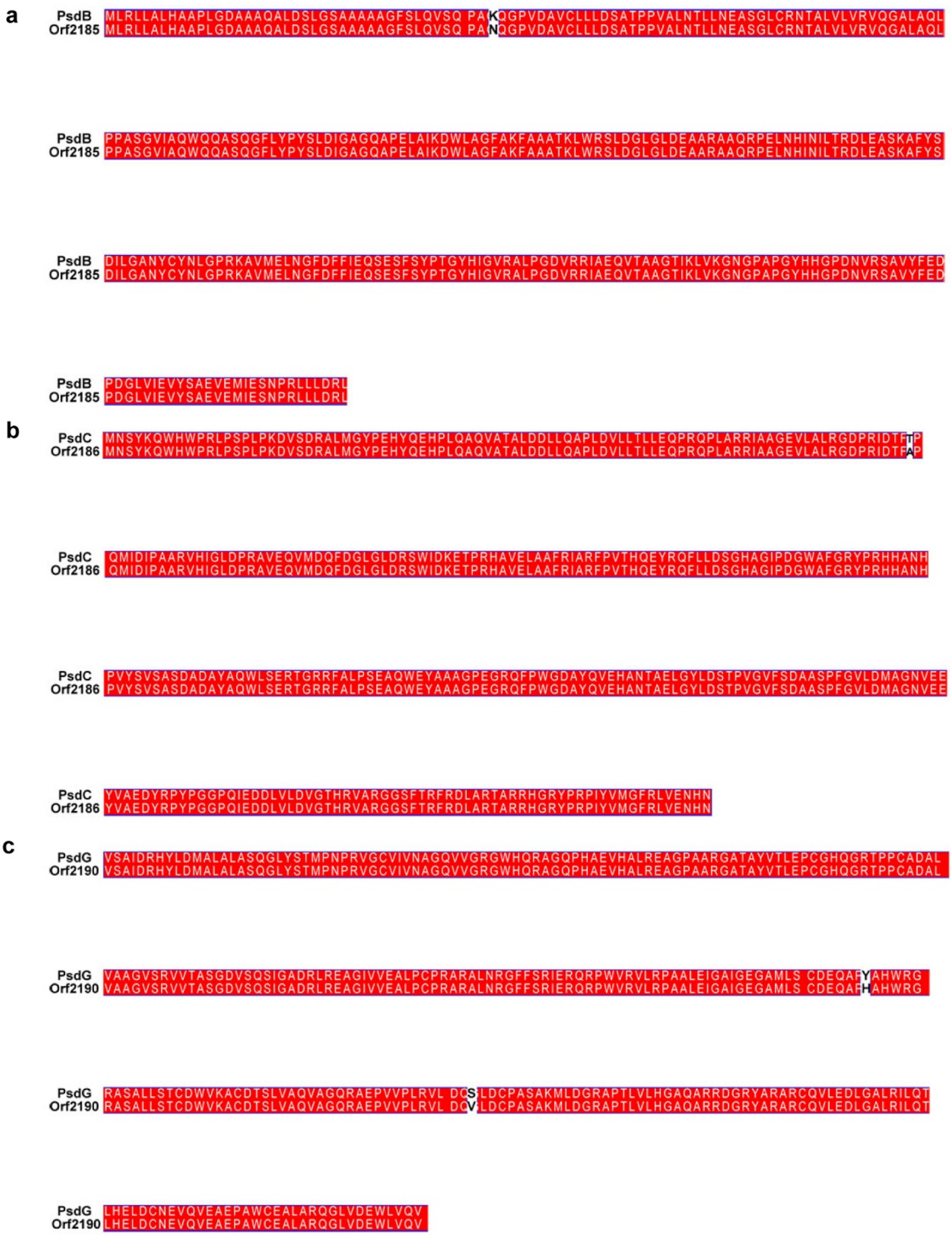

Supplementary Figure 16. Amino acid sequence alignments between proteins encoding pseudoiodinine in *P. mosselii* 923 (PsdB, PsdC and PsdG) and *P. mosselii* DSM17497 (Orf2185, Orf2186 and Orf2190). Alignments are shown for PsdB and Orf2185 (**a**), PsdC and Orf2186 (**b**) and PsdG and Orf2190 (**c**). Red-filled rectangles indicate the location of conserved residues and open, white rectangles indicate the location of different amino acid residues in the *P. mosselii* 923 and DSM17497 gene clusters.

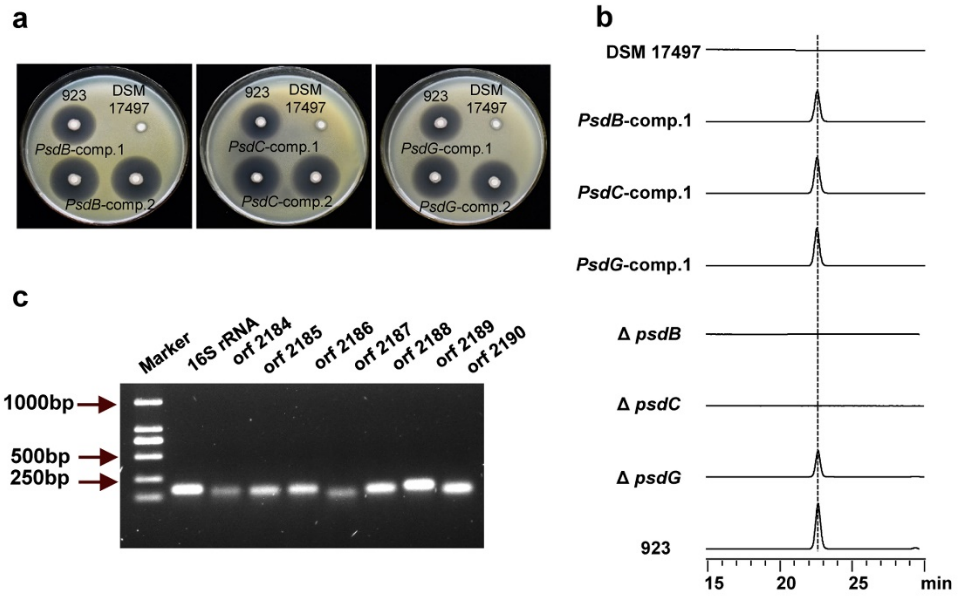

Supplementary Figure 17. Characterization of the pseudoiodinine gene cluster in *P. mosselii* DSM17497. a, Antimicrobial activity of *P. mosselii* 923, *P. mosselii* DSM17497, and two complemented strains each for Δ*psdB*, Δ*psdC*, and Δ*psdG* mutants (*PsdB*-comp.1, *PsdB*-comp.2, *PsdC*-comp.1, *PsdC*-comp.2, *PsdG*-comp.1 and *PsdG*-comp.2). **b,** HPLC profiles (Method B, detected at 500 nm) of *P. mosselii* 923, *P. mosselii* DSM17497, 923 Δ*psdB*, Δ*psdC*, Δ*psdG* mutants and 923 complemented strains containing the DSM17497 *psd* operon. **c,** RT-PCR verification of the expression of *16S rRNA* and *orf2184* (*psdA*); *orf2185* (*psdB*); *orf2186* (*psdC*); *orf2187* (*psdD*); *orf2188* (*psdE*); *orf2189* (*psdF*) and *orf2190* (*psdG*) in *P. mosselii* DSM17497. Parentheses indicate the homologous genes in *P. mosselii* 923. Similar results were observed in three independent experiments.

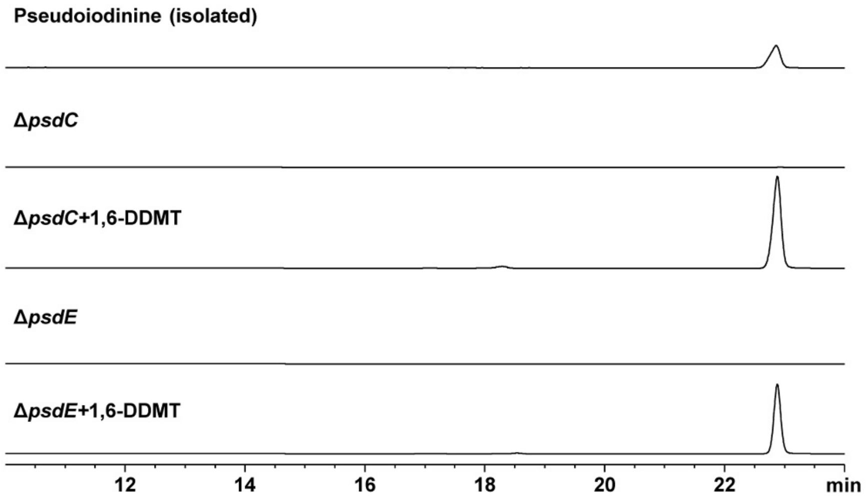

Supplementary Figure 18. HPLC analysis (A_{500}) of pseudoiodinine production in the mutant strains Δ*psdC* and Δ*psdE* that were fed with 1,6-DDMT, respectively.

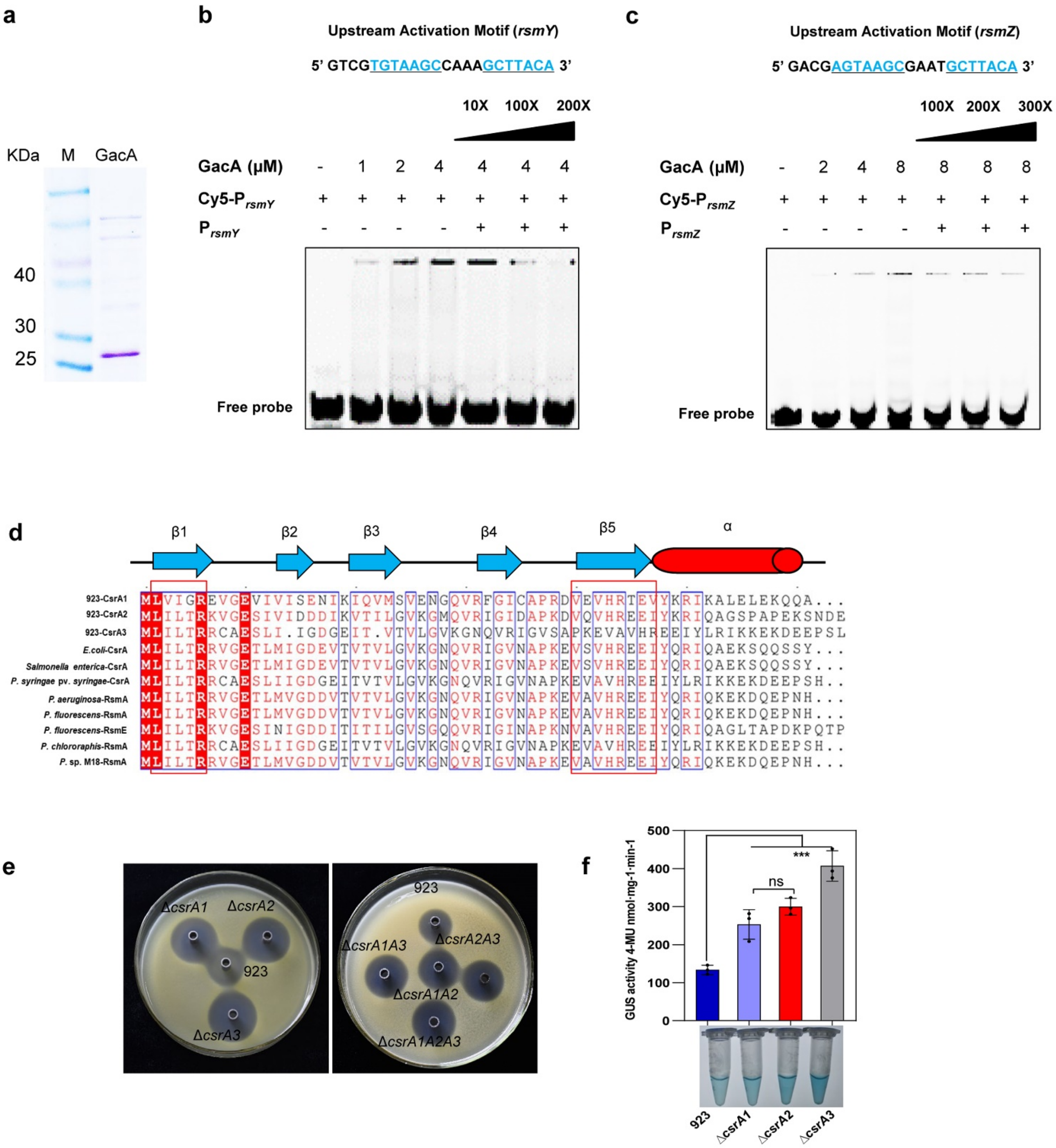
a
KDa M GacA
40
30
25
b
Upstream Activation Motif (rsmY)
5' GTCGTGTAAGCCAAAGCTTACA 3'
10X 100X 200X
GacA (μM) - 1 2 4 4 4 4
Cy5-PrsmY + + + + + + +
PrsmY - - - - + + +
Free probe
c
Upstream Activation Motif (rsmZ)
5' GACGAGTAAGCGAATGCTTACA 3'
100X 200X 300X
GacA (μM) - 2 4 8 8 8 8
Cy5-PrsmZ + + + + + + +
PrsmZ - - - - + + +
Free probe
d
β1 β2 β3 β4 β5 α
923-CsrA1
923-CsrA2
923-CsrA3
E.coli-CsrA
Salmonella enterica-CsrA
P. syringae pv. syringae-CsrA
P. aeruginosa-RsmA
P. fluorescens-RsmA
P. fluorescens-RsmE
P. chlororaphis-RsmA
P. sp. M18-RsmA
e
ΔcsrA1 ΔcsrA2
923
ΔcsrA3
923
ΔcsrA1A3 ΔcsrA2A3
ΔcsrA1A2
ΔcsrA1A2A3
f
GUS activity 4-MU nmol·mg-1·min-1
500
400
300
200
100
0
***
ns
923
ΔcsrA1
ΔcsrA2
ΔcsrA3

Supplementary Figure 19. Identification of regulatory genes for pseudoiodinine biosynthesis in *P. mosselii* 923. a, Purification of His-GacA fusion protein. **b, c**, EMSA experiments assessing the binding of the GacA protein to Cy5-labeled *rsmY* (**b**) and *rsmZ* (**c**) promoter fragments (probes). Positions of the bound and free probe are indicated at the left. In panel (**b**), the concentration of GacA increased from 0, 1, 2, and 4 μM and the final concentration of Cy5-P*rsmY* probe was 0.1 μM, with competitive concentrations of P*rsmY* in the order of 10x, 100x and 200x. In panel (**c**) the concentration of GacA increased in the order of 0, 2, 4, and 8 μM and the final concentration of Cy5-P*rsmZ* probe was 0.1 μM; the competitive concentration of P*rsmZ* was 100x, 200x and 300x. **d,** Protein secondary structure and multiple sequence alignments for CsrA, RsmA, and RsmE orthologs in *P. mosselii* 923 and other bacterial species. β-strands and α-helices in the proteins are depicted as blue arrows and red cylinders, respectively. Red rectangular boxes indicate the location of conserved residues that are important for RNA binding and in vivo regulation**. e,** Antibacterial activity assays of *P. mosselii* 923 and *csrA1*, *csrA2*, *csrA3*, *csrA1A2*, *csrA1A3*, *csrA2A3* and *csrA1A2A3* mutants. **f**, Expression of *psdA* promoter-driven β-glucuronidase (GUS) activity in *P. mosselii* 923 and the Δ*csrA1*, Δ*csrA2*, Δ*csrA3* mutants in LB medium. Error bars show means ± SD (n = 3 biological independent replicates) and significant differences at ***P < 0.001, ***P= 0.001, = 0.0002, = 0.000004 in sequence, ns, not significant (P > 0.05) (one-way ANOVA followed by LSD test). Experiments were performed three times independently with similar results.

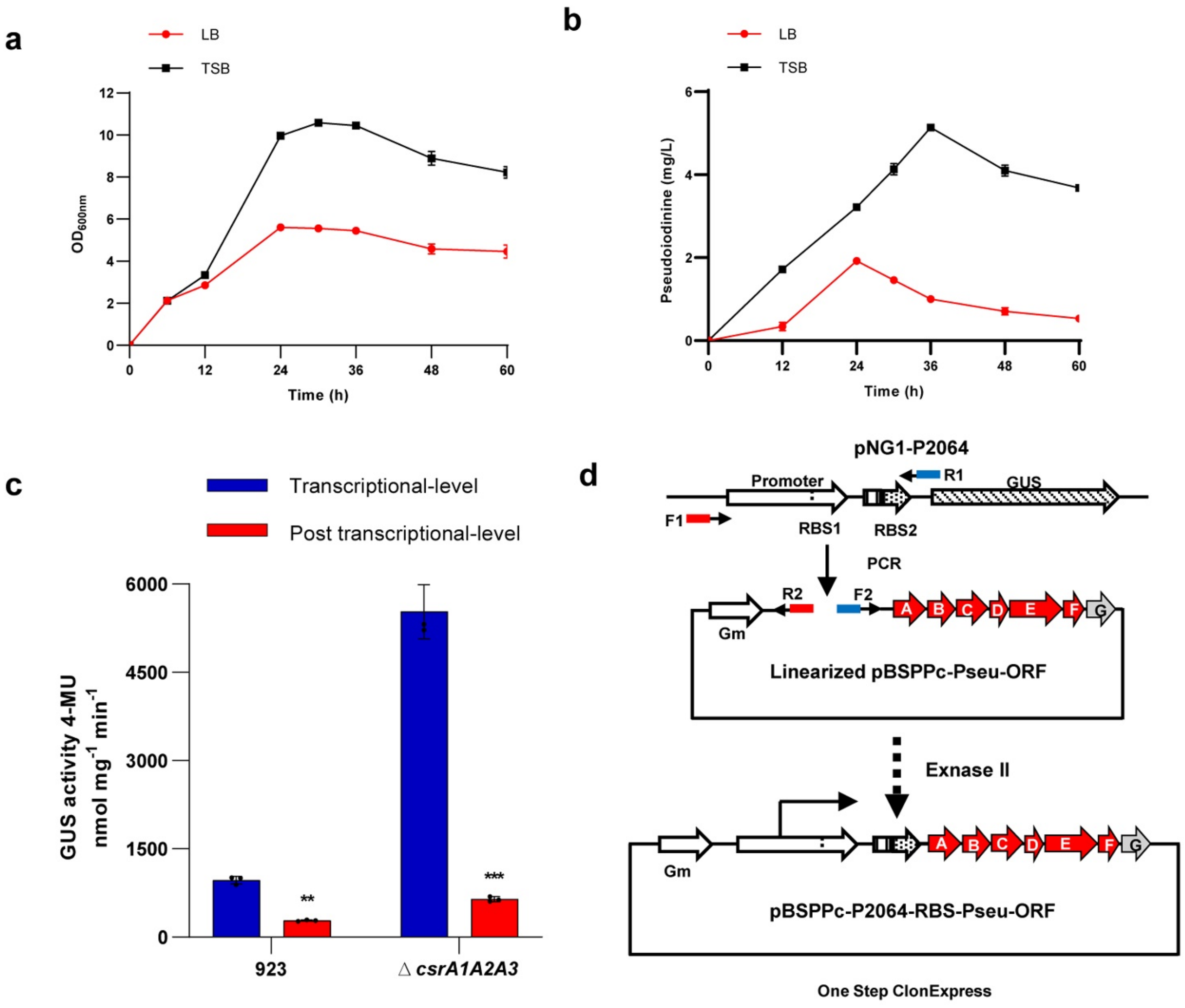

Supplementary Figure 20. Optimizing pseudoiodinine production. a, b, Effect of medium on growth (**a**) and pseudoiodinine production (**b**) by *P. mosselii* 923 in LB and TSB media. Error bars represent standard deviations of three biological independent duplicates (means ± SD, n = 3). **c**, The RBS sequence-driven β-glucuronidase (GUS) activity of 923 and the Δ*csrA1A2A3* mutant at the transcriptional and post-transcription level. Error bars show means ± SD (n = 3 biological independent replicates) and significant differences at $^{**}P < 0.01$, $^{***}P < 0.001$, $^{**}P = 0.007$, $^{***}P = 5.7\times10^{-9}$ (one-way ANOVA followed by LSD test). Experiments were performed three times independently with similar results. **d**, Schematic diagram showing construction of the pBSPPc-P2064-RBS-Pseu-ORF plasmid using One Step ClonExpress technology.

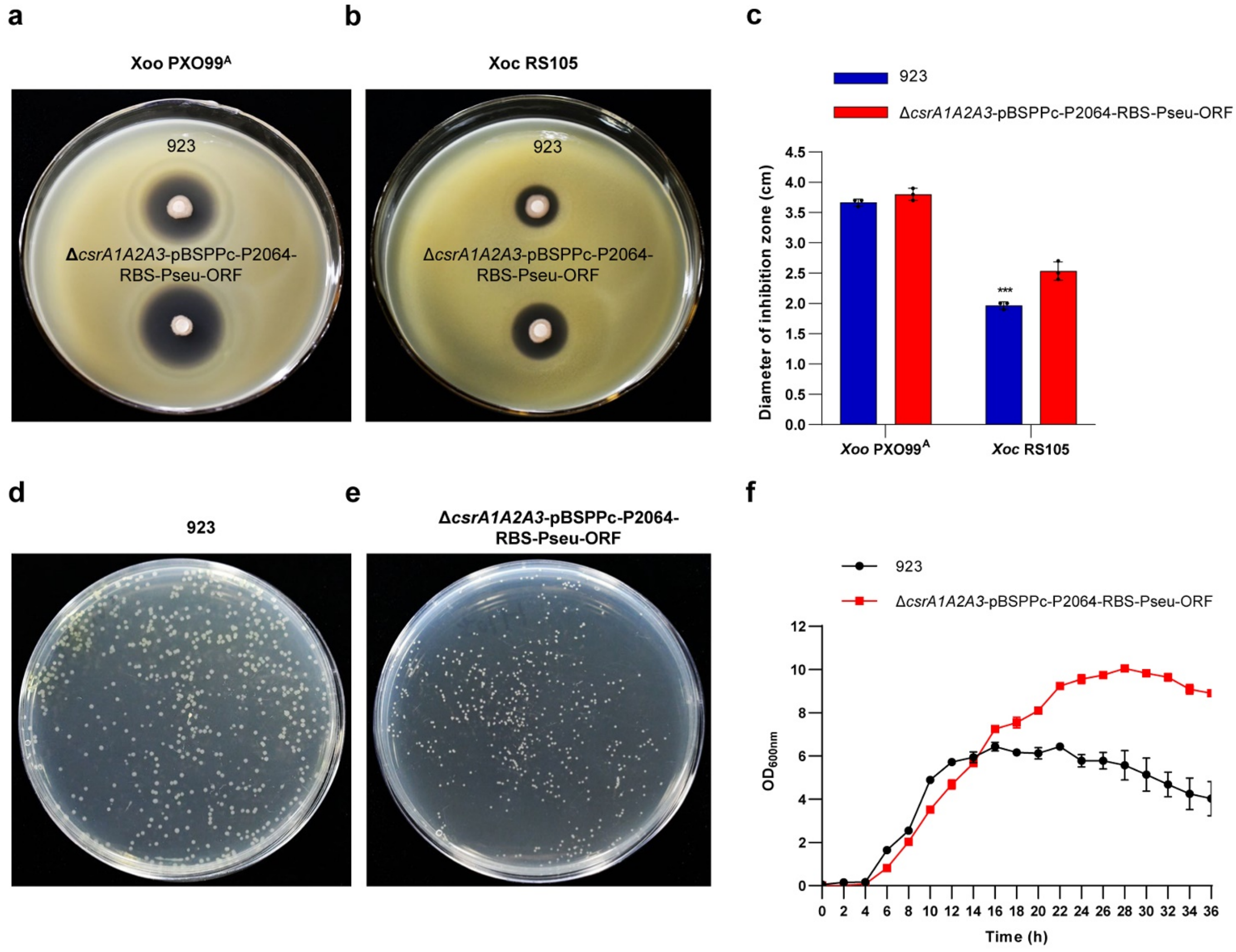

Supplementary Figure 21. Antimicrobial and growth analysis. Antimicrobial activity of *P. mosselii* 923 and Δ*csrA1A2A3*-pBSPPc-P2064-RBS-Pseu-ORF strains for (**a**) *Xoo* PXO99^{A} and (**b**) *Xoc* RS105. **c**, The diameter of inhibition zones ± SD (cm) (n=3 biologically independent plates) and significant differences at ***P < 0.001, ***P= 0.0001 (one-way ANOVA followed by LSD test). Growth of strains (**d**) *P. mosselii* 923 and (**e**) Δ*csrA1A2A3*-pBSPPc-P2064-RBS-Pseu-ORF on LB medium with 10^{-5} dilution of TSB. **f**, Growth of *P. mosselii* 923 and Δ*csrA1A2A3*-pBSPPc-P2064-RBS-Pseu-ORF in TSB medium. Samples were taken every 2 h for cell density (OD_{600}) measurements. Error bars represent standard deviations of three biologically independent samples (mean ± SD, n = 3). Three independent biological experiments were performed with similar results.

Supplementary Tables

Supplementary Table 1. X-ray Crystallographic Data for PM-3 (pseudoiodinine).

**Identification code**	**Pseudoiodinine**	
Empirical formula	C12 H21.22 N10 O5	
Formula weight	385.60	
Temperature	169.99(15) K	
Wavelength	1.54184 Å	
Crystal system	Monoclinic	
Space group	P 1 21/c 1	
Unit cell dimensions	a = 17.3087(4) Å	α= 90°
	b = 6.5227(2) Å	β= 94.743(2)°
	c = 15.5548(4) Å	γ = 90°
Volume	1750.11(8) $Å^3$	
Z	4	
Density (calculated)	1.463 Mg/m^3	
Absorption coefficient	0.993 mm^{-1}	
F(000)	813	
Crystal size	0.15 x 0.02 x 0.01 mm^3	
Theta range for data collection	5.128 to 75.442°.	
Index ranges	-20<=h<=21, -7<=k<=8, -19<=l<=19	
Reflections collected	11848	
Independent reflections	3469 [R(int) = 0.0562]	
Completeness to theta = 67.684°	99.9 %	
Absorption correction	Semi-empirical from equivalents	
Max. and min. transmission	1.00000 and 0.58436	
Refinement method	Full-matrix least-squares on F^2	
Data / restraints / parameters	3469 / 48 / 387	
Goodness-of-fit on F^2	1.036	
Final R indices [I>2sigma(I)]	R1 = 0.0612, wR2 = 0.1586	
R indices (all data)	R1 = 0.1013, wR2 = 0.1946	
Extinction coefficient	n/a	
Largest diff. peak and hole	0.335 and -0.449 e.$Å^{-3}$	

The crystallographic data for **PM-3** (pseudoiodinine) have been deposited at the Cambridge Crystallographic Data Center (deposition no. CCDC 2175188). Copies of the data can be obtained free of charge via www.ccdc.cam.ac.uk or from the Cambridge Crystallographic Data Center, 12 Union Road, Cambridge CB2 1EZ, UK [fax: +44(0) (1223) 336 033; e-mail: deposit@ccdc. cam.ac.uk].

Supplementary Table 2. DEGs that are significantly downregulated in the *ΔgacA* mutant as compared to the wild-type *P. mosselii* 923.

ID	log2 fold change	Description
chr_3630	-2.975614347	Hydroxymethylglutaryl-CoA synthase
chr_3632	-1.541914089	Asparagine synthetase
chr_2060	-7.323943531	RND efflux system, outer membrane lipoprotein
chr_2070	-5.42232076	Riboflavin biosynthesis protein RibD
chr_2062	-1.927759127	Nonribosomal peptide synthetase
chr_3132	-2.44272608	Flavin containing amine oxidoreductase
chr_2055	-1.377188789	Bacteriophage N4 receptor, outer membrane subunit
chr_2073	-1.208738466	UPF0145 protein
chr_2056	-1.062244436	Type II secretion system protein E
chr_3639	-1.455062748	Transposase (IS4 family) protein
chr_3643	-1.545988033	Transposase
chr_3636	-1.251095812	FMN-dependent NADH-azoreductase
chr_3637	-1.089077197	Glutathione S-transferase
chr_2173	-3.049957091	Flavin reductase domain protein, FMN-binding
chr_3133	-2.668401838	Decarboxylase
chr_2057	-1.454478613	UDP-N-acetylglucosamine 2-epimerase
chr_3621	-1.014686009	Sulfate ABC transporter periplasmic sulfate-binding protein
chr_2064	-4.084253308	Methyltransferase type 12
chr_2071	-4.787626863	Methyltransferase
chr_2072	-3.920655605	Major facilitator superfamily
chr_2171	-3.39502825	Domain of unknown function (DUF1852)
chr_2172	-3.412329848	5-methyltetrahydropteroyltriglutamate--homocysteine methyltransferase
chr_3629	-3.103403512	Transcriptional regulator, LysR family
chr_2065	-5.809440432	Glyoxylase bleomycin resistance protein dioxygenase superfamily protein
chr_2066	-6.70502851	Sulphatase-modifying factor protein
chr_2061	-7.367696232	Transcriptional regulator, LuxR family
chr_2069	-7.181850911	Methyltransferase domain
chr_2067	-8.293903239	GTP cyclohydrolase
chr_2068	-7.3355698	WD-40 repeat protein

Supplementary methods

β-glucuronidase (GUS) assays.

To quantify GUS activity in rice, the inoculated rice leaves were collected, frozen in liquid nitrogen and then ground using the tissue grinder (JX-FSTPRP) at 55 Hz for 30 s. 1 mL GUS extraction buffer (50 mM phosphate buffer pH 7.0, 10 mM EDTA pH 8.0, 20% methanol, 0.1% SDS, 0.1% β-mercaptoethanol and 0.1% Triton X-100) was added and mixed thoroughly. Then centrifuged at 4 °C 13,800*g* for 20 min, 10 μL supernatant was added to 90 μL assay buffer (50 mM sodium phosphate, pH 7.0, 1 mM 4-methylumbelliferyl β-D-glucuronide (4-MUG), 10 mM β-mercaptoethanol and 0.1% Triton X-100), and incubated at 37 °C for 1 h. Finally, 900 μL 0.2 M Na_2CO_3 was added to terminate this reaction. 200 μL of above reaction solution was measured by a fluorescence spectrophotometer (GloMax Multi Detection System, Promega). The GUS activity was calculated according to the following formula: Y = (X + 23,751)/5,942, this was a standard curve with 4-methylumbelliferone (4-MU), X represented the measured UV value, Y represented the value of enzyme activity, which was calculated in $U \cdot OD_{600}^{-1} \cdot min^{-1}$. The unit of U is 4-MU $nmol \cdot mg^{-1} \cdot min^{-1}$. For qualitative GUS assays, the rice leaves were stained for 12 h using X-gluc staining solution from gusblue kit (Huayueyang, Beijing, China, Catalog No.GT0391), then decoloured by 75% alcohol. The experiments were repeated three times independently.

To quantify GUS activity in *P. mosselii*, the bacterial cells were mixed with 500 μL 2× Sonic buffer (20 mM Tris-HCl, 10 mM β-mercaptoethanol, 5 mM EDTA and 1% Triton X-100), then frozen in liquid nitrogen for 3 min and incubated at 37 °C for 5 min, which was repeated six times. 10 μL of supernatant from centrifugation at 4 °C 13,800*g* for 20 min was added to 90 μL assay buffer (37 °C, 1 h). The terminate reaction and GUS activity were processed following the above methods. For

qualitative GUS assays, the bacterial cells were stained for 1 h using staining solution (50mg X-gluc dissolved in 1mL N, N-dimethylformamide), then taken photos. Three independent experiments were performed.
