# Supplementary material for: The natural pyrazolotriazine pseudoiodinine from Pseudomonas mosselii 923 inhibits plant bacterial and fungal pathogens
Source: Nat Commun. 2023 Feb 9;14:734. doi: 10.1038/s41467-023-36433-z (PMC9911603; doi:10.1038/s41467-023-36433-z)
Supplement: Supplementary file 3 — Description of Additional Supplementary Files [file 41467_2023_36433_MOESM3_ESM.pdf]

Description of Additional Supplementary Files

Supplementary Data 1: Strains and plasmids used in the current study.

Supplementary Data 2: Primers used in this study.
