# Supplementary material for: The natural pyrazolotriazine pseudoiodinine from Pseudomonas mosselii 923 inhibits plant bacterial and fungal pathogens
Source: Nat Commun. 2023 Feb 9;14:734. doi: 10.1038/s41467-023-36433-z (PMC9911603; doi:10.1038/s41467-023-36433-z)
Supplement: Supplementary file 5 — Reporting Summary [file 41467_2023_36433_MOESM5_ESM.pdf]

nature portfolio

Corresponding author(s): Gongyou Chen,Lifang Zou

Last updated by author(s): Jan 31, 2023

Reporting Summary

Nature Portfolio wishes to improve the reproducibility of the work that we publish. This form provides structure for consistency and transparency in reporting. For further information on Nature Portfolio policies, see our Editorial Policies and the Editorial Policy Checklist.

Statistics

For all statistical analyses, confirm that the following items are present in the figure legend, table legend, main text, or Methods section.

n/a	Confirmed	
[ ]	[x]	The exact sample size (*n*) for each experimental group/condition, given as a discrete number and unit of measurement
[ ]	[x]	A statement on whether measurements were taken from distinct samples or whether the same sample was measured repeatedly
[ ]	[x]	The statistical test(s) used AND whether they are one- or two-sided<br>*Only common tests should be described solely by name; describe more complex techniques in the Methods section.*
[x]	[ ]	A description of all covariates tested
[x]	[ ]	A description of any assumptions or corrections, such as tests of normality and adjustment for multiple comparisons
[ ]	[x]	A full description of the statistical parameters including central tendency (e.g. means) or other basic estimates (e.g. regression coefficient) AND variation (e.g. standard deviation) or associated estimates of uncertainty (e.g. confidence intervals)
[ ]	[x]	For null hypothesis testing, the test statistic (e.g. *F*, *t*, *r*) with confidence intervals, effect sizes, degrees of freedom and *P* value noted<br>*Give P values as exact values whenever suitable.*
[x]	[ ]	For Bayesian analysis, information on the choice of priors and Markov chain Monte Carlo settings
[x]	[ ]	For hierarchical and complex designs, identification of the appropriate level for tests and full reporting of outcomes
[x]	[ ]	Estimates of effect sizes (e.g. Cohen's *d*, Pearson's *r*), indicating how they were calculated

Our web collection on statistics for biologists contains articles on many of the points above.

Software and code

Policy information about availability of computer code

Data collection	1.Type Genome Server (TYGS) (https://tygs.dsmz.de); 2. antiSMASH program (https://antismash.secondarymetabolites.org/);3. J Species WS online service (https://jspecies.ribohost.com/jspeciesws/#anib); 4. Heatmaps were produced by R language and the Pheatmap software package (https://rdrr.io/cran/pheatmap/); 5. Volcano plots were created using the R language ggplots2 package and plot_volcano from soothsayer (https://github.com/jolespin/soothsayer) in Python v. 3.6.6; 6. Bruker Avance III 600M spectrometer (Germany); 7.Agilent UPLC 1290 Infinity II/6545 Q-TOF (USA); 8.Agilent Technologies 1260 Infinity II HPLC with a DAD detector; 9. Illumina HiSeq system. DESeq v. 1.18.0; 10.an ABI7500 Real-Time PCR System (Applied Biosystems, USA); 11. a tissue grinder (JX-FSTPRP) at 55 Hz; 12. a Nano-300 MicroSpectrophotometer (Hangzhou Allsheng Instruments Co.); 13. an Amersham Typhoon RGB Biomolecular Imager (Cytiva, Sweden); 14. a fluorescence spectrophotometer (GloMax Multi Detection System, Promega).
Data analysis	1. Statistical significance was calculated using the least significant difference (LSD) test method and one-way ANOVA analysis for multiple comparisons using SPSS v. 22.0. 2. The statistical tests value represent statistically significant in the figure legends as follows: *P < 0.05; **P < 0.01; ***P < 0.001, one-way ANOVA followed by LSD test. 3. Data are presented as means ± standard deviation (SD). 4. Statisticals data were analyzed using GraphPad Prism version 8.00. 5. Lesions areas were calculated from infected leaves using Adobe Photoshop CS5.

For manuscripts utilizing custom algorithms or software that are central to the research but not yet described in published literature, software must be made available to editors and reviewers. We strongly encourage code deposition in a community repository (e.g. GitHub). See the Nature Portfolio guidelines for submitting code & software for further information.

Data

Policy information about availability of data

All manuscripts must include a data availability statement. This statement should provide the following information, where applicable:

- Accession codes, unique identifiers, or web links for publicly available datasets
- A description of any restrictions on data availability
- For clinical datasets or third party data, please ensure that the statement adheres to our policy

The data that support the findings of this study are available within this manuscript and its Supplementary Information file. The genome sequence data of Pseudomonas mosselii 923 used in this study has been deposited in the NCBI GenBank database under the BioProject accession code PRJNA826312 (https://www.ncbi.nlm.nih.gov/nuccore/NZ_CP095556). The transcriptome data has been deposited to the NCBI Sequence Read Archive and is accessible with code PRJNA880759 (https://www.ncbi.nlm.nih.gov/bioproject/PRJNA880759). Crystallographic data for the structure of pseudoiodinine reported in this Article has been deposited at the Cambridge Crystallographic Data Centre, under deposition number CCDC 2175188. Copies of the data can be obtained free of charge via www.ccdc.cam.ac.uk. Strains, plasmids and primers used in this study are reported in the Supplementary Dataset. Source data are provided with this paper. Data is also available from the corresponding author upon request.

Human research participants

Policy information about studies involving human research participants and Sex and Gender in Research.

Reporting on sex and gender	not applicable
Population characteristics	not applicable
Recruitment	not applicable
Ethics oversight	not applicable

Note that full information on the approval of the study protocol must also be provided in the manuscript.

Field-specific reporting

Please select the one below that is the best fit for your research. If you are not sure, read the appropriate sections before making your selection.

☒ Life sciences ☐ Behavioural & social sciences ☐ Ecological, evolutionary & environmental sciences

For a reference copy of the document with all sections, see nature.com/documents/nr-reporting-summary-flat.pdf

Life sciences study design

All studies must disclose on these points even when the disclosure is negative.

Sample size	Sample size was not predetermined by statistical method as this study did not involve animal models or human subjects. Sample sizes were chosen based on the Authors' experience of what is necessary to generate a convincing and compelling result. Sample sizes with respect to the number of independent experiments are indicated in the respective figure legends. For all assays sample size was determined in advance and was uniform .
Data exclusions	No data were excluded from our analyses.
Replication	In a parallel experiment, we set up triple replications for determining the mean value and standard deviation. All attempts at replication were successful. All the experiments were conducted at least three times to verify the reproducibility.
Randomization	Samples were randomly allocated into experimental groups. The soil samples were randomly collected from the rhizosphere.
Blinding	Blinding is not applicable in our study because there were no need to allocate samples into experimental groups. Investigators were not blinded to group allocation during data collection and analysis. Because it was not relevant to most of the experiments carried out and these were based on molecular genetic and natural product characteristics, which required knowledge of samples to be processed. Our experiment results are not subjective.

Reporting for specific materials, systems and methods

We require information from authors about some types of materials, experimental systems and methods used in many studies. Here, indicate whether each material, system or method listed is relevant to your study. If you are not sure if a list item applies to your research, read the appropriate section before selecting a response.

Materials & experimental systems

n/a	Involved in the study
☒	☐ Antibodies
☒	☐ Eukaryotic cell lines
☒	☐ Palaeontology and archaeology
☒	☐ Animals and other organisms
☒	☐ Clinical data
☒	☐ Dual use research of concern

Methods

n/a	Involved in the study
☒	☐ ChIP-seq
☒	☐ Flow cytometry
☒	☐ MRI-based neuroimaging
